# Histone modification clocks for robust cross-species biological age prediction and elucidating senescence regulation

**DOI:** 10.1073/pnas.2533687123

**Published:** 2026-03-10

**Authors:** Zhixin Niu, Chang Liu, Yurong Fan, Lei Gu

**Affiliations:** ^a^Epigenetics Laboratory, Max Planck Institute for Heart and Lung Research & Cardiopulmonary Institute, Bad Nauheim 61231, Germany; ^b^Faculty of Medicine, Justus-Liebig-University Giessen, Giessen 35392, Germany

**Keywords:** epigenetics, histone modification, aging

## Abstract

Histone modifications represent an untapped resource for biological age prediction that overcomes limitations of traditional DNA methylation-based epigenetic clocks. Here, we developed and validated histone modification-based epigenetic clocks by systematically analyzing publicly available ChIP-seq datasets spanning six tissue types and six histone marks. We identified age-associated loci and constructed 36 tissue-specific epigenetic clocks that demonstrated strong resilience to technical and biological noise, with performance comparable to established DNA methylation clocks. Our models successfully detected biological age acceleration in leukemia samples and captured age reversal following therapeutic interventions. Importantly, we found that many aging-associated loci follow nonlinear trajectories with peak modification levels at midlife, revealing previously unrecognized dynamics in epigenetic aging. We observed age-related fragmentation of super enhancer regions, suggesting progressive chromatin disorganization during aging. Functional validation of a model-selected H3K27ac peak near IGF2BP3 confirmed its causal role in cellular senescence through regulation of TRA2A expression. Extending beyond mammals, we demonstrated the applicability of histone-based clocks in *Drosophila melanogaster*, a species lacking DNA methylation, highlighting the evolutionary conservation and broader utility of histone modifications as aging biomarkers. Our findings establish histone modifications as accurate, biologically meaningful, and robust indicators of biological age with potential applications in aging research, disease monitoring, and therapeutic development across diverse species.

## Significance

While DNA methylation is the standard for epigenetic aging, histone modifications regulate the dynamic plasticity of the genome yet remain an untapped resource for age prediction. We developed robust histone-based clocks that parallel methylation performance but offer distinct mechanistic insights. Our study reveals that chromatin aging involves nonlinear trajectories and the fragmentation of super-enhancers-structural dynamics missed by linear models. We demonstrate these clocks’ utility in detecting age acceleration in leukemia and capturing therapeutic age reversal. Uniquely, we validate this approach in *Drosophila*, establishing evolutionary conservation where DNA methylation clocks fail. This work positions histone modifications as a critical, biologically rich dimension of the aging epigenome, essential for understanding disease mechanisms and monitoring rejuvenation.

Chronological age, the number of years a person has lived, represents one of the strongest risk factors for mortality and age-associated diseases (1, 2). However, this simple metric fails to capture the complex physiological changes that characterize the aging process. Biological age, in contrast, provides a more nuanced assessment of an individual’s health status and functional capacity by measuring molecular and cellular hallmarks of aging (3, 4). This distinction is critical because individuals of identical chronological age often display markedly different biological ages due to diverse lifestyle factors including diet (5–7), physical activity (6, 8), environmental exposures (6, 8), and disease states (9–11). The discrepancy between biological and chronological age, termed age acceleration when biological age exceeds chronological age, has emerged as a powerful predictor of health outcomes, with accelerated aging associated with increased risk of chronic diseases and mortality, while decelerated aging correlates with improved health and longevity (8, 12–14).

Epigenetic clocks have emerged as among the most accurate methods for estimating biological age (15). These predictive models, primarily based on DNA methylation, can estimate chronological age with remarkable precision and predict age-related health outcomes (16–18). Beyond DNA methylation, researchers have developed biological age predictors using various molecular data types, including proteomics (19–21), metabolomics (22–24), transcriptomics (25, 26), microbiome profiles (27), glycomics (28, 29), and chromatin accessibility (30). Each approach captures different aspects of the aging process, contributing to a multidimensional understanding of biological aging.

However, DNA methylation is not always functionally linked to gene expression, complicating efforts to infer downstream biological consequences from predictive CpG sites. In addition, several important model organisms, such as *D. melanogaster*, lack canonical DNA methylation systems (31, 32), which precludes the application of traditional epigenetic clocks in these species. Thus, it motivates the search for alternative aging biomarkers more directly connected to transcriptional regulation and broad application.

Alternative epigenetic marks, particularly histone modifications, offer potential solutions to these limitations. Histone modifications play crucial roles in regulating chromatin structure and gene expression, and their patterns change during aging (33–39). Notably, heterochromatin stability, a key hallmark of aging, is maintained by repressive histone modifications that silence transposable elements and preserve genomic integrity. Furthermore, the evolutionary conservation of histone modifications across species provides an opportunity to develop aging biomarkers applicable to organisms lacking DNA methylation systems. These features suggest that histone modifications may both reflect and influence aging biology.

Therefore, we investigated whether histone modifications can serve as reliable biomarkers for constructing epigenetic clocks. We curated and processed ChIP-seq datasets of six histone marks across six human tissues from healthy donors spanning a wide age range. Using these data, we identified age-associated histone peaks and trained elastic net regression models to predict biological age. The resulting clocks exhibited strong predictive performance (mean absolute error of 4.67 ± 3.73 y) and highlighted specific histone features that may actively participate in aging regulation. Notably, knockdown of model-identified H3K27ac peaks in vitro induced cellular senescence phenotypes, suggesting causal roles for selected chromatin elements. Finally, we validated the utility of histone modification-based clocks in *D. melanogaster*, a species lacking canonical DNA methylation, reinforcing the evolutionary conservation of this approach.

Together, our study expands the landscape of epigenetic aging biomarkers by establishing histone modifications as accurate, biologically meaningful, and mechanistically informative indicators of biological age.

## Results

### Mapping Age-Associated Histone Modification Landscapes across Tissues and Marks.

**Fig. 1.**
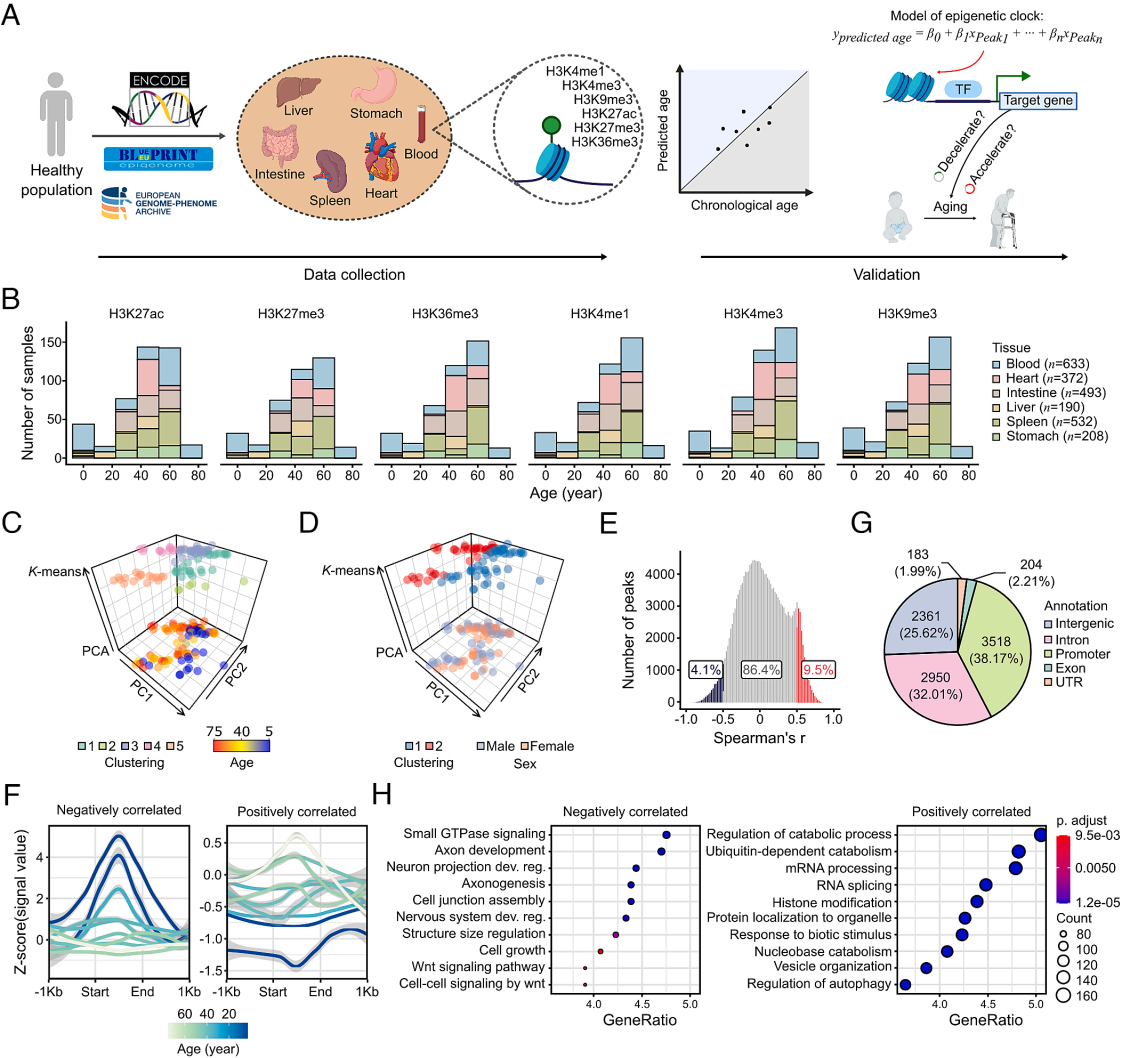
(*A*) Schematic of developing and verifying the histone modification-based epigenetic clocks. The workflow begins with data collection. Then, consensus peak sets containing signal values for each sample were identified, followed by identifying age-correlated peaks. Then, modeling is done through the application of an elastic net regression for feature selection. Peaks identified as relevant to the aging process are further validated for their biological significance. Created with BioRender.com. (*B*) Sample distribution by chronological age for histone modifications across tissues. (*C*) Clustering of blood H3K27ac samples by age. A 3D spatial plot illustrates K-means clustering into five age groups (upper layer), alongside a PCA-based dimension reduction with samples colored by their chronological ages (lower layer). (*D*) Similar to panel (*C*), this plot shows PCA clustering with samples colored by sex, indicating gender has minimal influence on sample distribution. (*E*) Correlation between H3K27ac peaks and age in blood samples. Peaks were called using MACS2, using matched input chromatin DNA (no antibody immunoprecipitation) from the same tissue as background controls. The histogram presents the distribution of Spearman’s r, with positively correlated peaks (Spearman’s r ≧ 0.5, *P* ≦ 0.05) in red and negatively correlated peaks (Spearman’s r ≦ −0.5, *P* ≦ 0.05) in blue. (*F*) Profile of H3K27ac signal across age groups. Signal intensity for negatively correlated peaks (*Upper*) and positively correlated peaks (*Lower*) are displayed relative to the peak center (±1 kb). Z-scored signal values are shown, with darker lines representing younger ages. (*G*) Genomic annotation of age-associated H3K27ac peaks in blood reveals their distribution across intergenic, intronic, promoter, exonic, and UTR regions, with a significant enrichment in promoter regions (Fisher’s exact test, *P* < 2.2e-16). (*H*) GO enrichment analysis of genes linked to H3K27ac peaks in blood that show negative (*Left*) and positive (*Right*) correlation with age. All terms presented were significantly enriched with adjusted *P* value less than 0.05.

To understand how the epigenetic landscape changes during aging, we established a comprehensive framework to systematically analyze histone modifications across human tissues (Fig. 1*A*). This approach allowed us to characterize age-associated epigenetic patterns and subsequently develop predictive models of biological age.

### Comprehensive Dataset of Histone Modifications across Tissues and Age Ranges.

We assembled an extensive collection of 2,428 histone ChIP-seq datasets from healthy individuals spanning from 5 to 75 y of age with a balanced sex distribution (54.6% male, 44.8% female, and 0.6% with missing information) (40–42). These datasets were derived from six distinct tissues: blood (*n* = 633), intestine (*n* = 493), spleen (*n* = 532), heart (*n* = 372), liver (*n* = 190), and stomach (*n* = 208) (Fig. 1*B*, *SI Appendix*, Fig. S1, and Dataset S1). For each tissue, we analyzed six key histone modifications representing both activating marks (H3K27ac, H3K4me1, H3K4me3, H3K36me3) and repressive marks (H3K27me3, H3K9me3). This dataset represents one of the largest collections of age-stratified histone modification profiles across multiple human tissues.

### Age-Associated Patterns in Histone Modifications Reveal Tissue-Specific Epigenetic Aging Signatures.

To identify age-associated epigenetic features, we first examined whether global histone modification patterns correlate with chronological age. Principal component analysis (PCA) of H3K27ac profiles in blood samples revealed a moderate correlation between the first principal component (PC1) and chronological age (Pearson’s correlation coefficient (r) = −0.49, *P* = 5.64e-07; Fig. 1*C*), indicating that age contributes continuously to epigenetic variation. In contrast, no apparent clustering or significant variation by sex was detected (Fig. 1*D*), suggesting that age exerts a stronger influence on the epigenetic landscape than sex in these samples. Similar age-associated clustering patterns were observed across other tissues and histone marks (*SI Appendix,* Figs. S2 and S3), with varying degrees of age-related separation.

We next quantified the relationship between individual histone modification peaks and chronological age. To ensure reliable peak identification, all ChIP-seq datasets were analyzed using matched input DNA controls generated from total chromatin without immunoprecipitation. In blood H3K27ac profiles, Spearman’s correlation coefficient (r) between H3K27ac signal and age was computed for 193,867 peaks. After Benjamini–Hochberg correction, no peaks achieved FDR < 0.05 (BH-adjusted *P*-values). Given the exploratory intent to nominate candidates for downstream validation, we applied a joint effect-size and nominal *P*-value filter (absolute Spearman’s r ≧ 0.5 and *P* ≦ 0.05), yielding 16,390 candidate peaks (10,989 positive, 5,401 negative correlations) (Fig. 1*E*). The distribution of correlation coefficients varied across tissues and histone marks (*SI Appendix,* Fig. S4; peak information available via Zenodo, see Data Availability), revealing tissue-specific patterns of age-associated epigenetic changes.

Visualization of signal profiles for age-associated H3K27ac peaks in blood revealed distinct patterns between positively and negatively correlated regions (Fig. 1*F*). Negatively correlated peaks showed progressive signal reduction with age, while positively correlated peaks exhibited increasing signal intensity. Importantly, these changes were not uniform across the genome but showed enrichment in specific genomic contexts. To determine whether age-associated H3K27ac peaks were preferentially located in promoter regions, we performed a Fisher’s exact test comparing the proportion of promoter-overlapping peaks in age-associated versus background peaks. The result showed a significant enrichment (Fisher’s exact test, *P* < 2.2e-16; Dataset S2), suggesting that age-related changes in histone modifications may directly impact gene expression programs (Fig. 1*G*). We next examined whether age-associated H3K27ac peaks are also enriched at enhancer regions. Age-associated H3K27ac peaks were significantly enriched at FANTOM5 predefined enhancer regions (Fisher’s exact test, *P* < 2.2e-16; Dataset S2), an effect driven predominantly by positively age-associated peaks, whereas negatively associated peaks showed no significant enhancer enrichment (Fisher’s exact test, *P* = 1; Dataset S2). This directional difference indicates that enhancer-associated H3K27ac changes during aging are not symmetric between gains and losses.

Functional annotation of age-associated peaks provided insights into the biological processes affected by these epigenetic changes. Gene Ontology (GO) analysis linked these peaks to mRNA processing, RNA splicing, histone modification, and autophagy regulation (Fig. 1*H*), highlighting functional relevance to aging biology. These enriched GO terms showed substantial overlap with pathways identified from age-associated differentially expressed genes (DEGs), including cell growth, mRNA processing, RNA splicing, and regulation of autophagy (Dataset S3). Age-associated peaks across tissues revealed clear tissue-specific functional enrichment patterns, with immune-related processes dominating blood and spleen, muscle, and cytoskeletal pathways enriched in the heart, epithelial morphogenesis in the intestine and stomach, and metabolic and proteostasis-related pathways enriched in the liver (Dataset S4).

### Nonlinear Trajectories of Histone Modifications Reveal Critical Transition Points in Aging.

When examining age-related changes in histone modification signals, we observed that many peaks followed nonlinear trajectories rather than simple linear relationships. Using locally estimated scatterplot smoothing (LOESS) regression, we identified inflection points in age-associated trajectories, particularly around ages 40 and 60 for H3K27ac in blood samples (*SI Appendix,* Fig. S5*A*). Similar nonlinear patterns were observed for other histone marks (*SI Appendix*, Fig. S5*A*), suggesting that epigenetic aging may proceed through discrete phases rather than as a continuous process (43). To investigate whether this pattern is presented across tissues, we merged H3K27ac data from all tissues into a single dataset. However, the previously observed inflection points were no longer evident (*SI Appendix*, Fig. S5*B*).

These nonlinear dynamics extended to higher-order chromatin structures. Analysis of super-enhancers which are clusters of enhancers that control cell identity genes revealed age-associated fragmentation, characterized by an increase in the number of super enhancers alongside a decrease in their average length with age (*SI Appendix*, Fig. S5 *C* and *D*). In contrast, typical enhancers showed coordinated changes in both number and length across age groups (*SI Appendix*, Fig. S5*E*). However, these patterns of super enhancers and typical enhancers cannot be identified from the merged dataset (*SI Appendix*, Fig. S5 *F* and *G*), suggesting the nonlinear signature is tissue-specific and may be masked and diluted when averaging across heterogeneous tissue types. This pattern suggests progressive disorganization of key regulatory domains during aging, potentially affecting transcriptional coordination and cellular identity maintenance.

Collectively, these findings establish a comprehensive atlas of age-associated histone modification changes across human tissues. These age-associated features provided the foundation for developing predictive models of biological age.

### Development and Validation of Histone Modification-Based Epigenetic Clocks.

To quantify the relationship between histone modifications and chronological age, we developed 36 tissue-specific epigenetic clocks using elastic net regression models. Each model was trained on normalized ChIP-seq peak signals and evaluated using a cross-validation framework. On average, models included approximately 80 nonzero coefficient features, indicative of discrete sets of informative loci per tissue and mark.

**Fig. 2.**
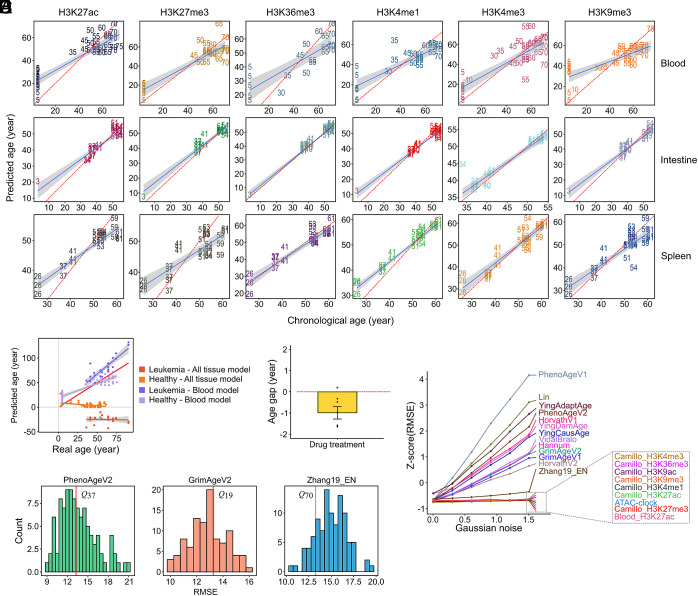
(*A*) Scatter plots of predicted age versus chronological age across tissues and histone modifications. Each panel illustrates the performance of epigenetic clocks constructed for blood, intestine, and spleen, based on six histone modifications. Each number indicates the chronological age (in years) of the corresponding sample. The red dashed line represents perfect concordance between predicted and chronological age, while the shaded areas denote the 95% CI for the regression lines. RMSE, MAE, and Pearson’s r values are reported in *SI Appendix*, Table S1. (*B*) Biological age between leukemia and healthy controls as predicted by the H3K27ac blood-specific model and the H3K27ac model trained across multiple tissues. (*C*) The scaled age gap, the difference between predicted biological age before and after treatment, was tested against zero using Student’s *t* test. (*D*) Distribution of RMSE values for age predictors trained on 100 randomly pooled sample sets from the ComputAgeBench dataset. The RMSE value of the histone mark-based predictor is indicated with a vertical-colored line, and its quantile position is annotated. (*E*) Evaluation of predictor robustness under noise across DNAm clocks, the ATAC-clock, Camillo’s histone mark-based clock, and the blood H3K27ac-based clock subjected to increasing levels of artificially added Gaussian noise. Data are presented as means ± SEM. Mann–Whitney *U* tests were performed for statistical analysis (*P < 0.05, **P < 0.01, ***P < 0.001).

The models achieved strong predictive performance, with a mean absolute error (MAE) of 4.67 ± 3.73 y, Rms error (RMSE) of 5.66 ± 4.38 y, and an average Pearson’s r of 0.91 ± 0.06 (Fig. 2*A* and *SI Appendix*, Fig. S6*A* and Table S1). Predictive power varied across tissues and marks, with H3K36me3 in liver and H3K27me3 in spleen performing less robustly likely due to sample heterogeneity and ChIP signal complexity. Since the clocks were trained on bulk ChIP-seq data, the resulting clock features should be interpreted at the tissue level and may reflect not only epigenetic aging within individual cell types but also age-associated changes in cellular composition.

To assess biological relevance, we applied the blood H3K27ac clock to leukemia patient samples. Compared to healthy controls, patients exhibited significant age acceleration, particularly in older individuals (Fig. 2*B*). However, the model trained on mixed tissue types did not capture age acceleration reliably, highlighting the critical role of tissue specificity in epigenetic age prediction (Fig. 2*B*). Moreover, in paired leukemia cell lines treated with anticancer compounds, predicted biological age was reduced in all cases, suggesting therapeutic reversal of epigenetic aging (Fig. 2*C*).

We next compared the performance of our histone modification-based clocks with existing epigenetic clocks. Using the ComputAgeBench framework (44), a benchmarking platform for evaluating diverse epigenetic clocks, we assessed our blood H3K27ac clock against established DNA methylation clocks under matched conditions. Our clock performed competitively, with its RMSE falling within the top quartile of the distribution of DNA methylation clock RMSEs (Fig. 2*D*). Similar results were observed for other tissue-modification combinations (*SI Appendix*, Fig. S6*B*).

A particularly notable advantage of our histone-based clocks was their resilience to technical and biological noise. When subjected to increasing levels of artificially added Gaussian noise, our blood H3K27ac clock maintained stable performance, with only modest degradation at high noise levels. In contrast, DNA methylation clocks showed more rapid performance deterioration under identical noise conditions (Fig. 2*E*). This enhanced robustness may reflect the broader, structural nature of histone modification signals compared to the more discrete CpG methylation sites, making histone-based clocks potentially more suitable for noisy or heterogeneous samples. Together, these analyses confirm that histone modification-based epigenetic clocks offer accurate and robust tools for estimating biological age and may possess unique advantages in clinical or heterogeneous data contexts.

Because our initial analyses were based on individual histone modifications, we next evaluated whether integrating multiple histone marks within the same tissue improves age prediction accuracy and robustness. To this end, in addition to the tissue-specific single-mark clocks, we generated cross-histone modification clocks by integrating all available histone modifications within each tissue. Prediction accuracy was evaluated using RMSE, MAE, and Pearson’s r. Compared with tissue-specific single-mark clocks, cross-histone modification clocks showed only limited improvements in RMSE and MAE, outperforming 13.9 to 34.9% of all tissue-specific single-mark models, but failing to exceed the best-performing single tissue–single mark clocks (*SI Appendix*, Fig. S7*A*). When evaluated using Pearson’s r, cross-histone modification clocks consistently underperformed most single-mark models, indicating a reduced ability to capture coherent age-associated trends across samples. To further examine whether combining complementary chromatin features could improve model performance, we additionally constructed clocks integrating two histone modifications with low genome-wide signal correlation, H3K27ac and H3K9me3, separately within the heart and blood. However, neither the heart nor the blood H3K27ac + H3K9me3 combined clock outperformed the corresponding tissue-specific single-mark clocks in terms of RMSE, MAE, or Pearson’s r (*SI Appendix*, Fig. S7*A*). We further assessed model robustness by introducing increasing levels of artificial Gaussian noise. Cross-histone modification clocks, including the heart and blood H3K27ac + H3K9me3 models, generally exhibited reduced robustness compared with tissue-specific single-mark clocks, as reflected by a more pronounced increase in relative RMSE under noise perturbation (*SI Appendix*, Fig. S7*B*). In contrast, tissue-specific single-mark clocks maintained more stable performance across noise levels. Together, these results indicate that integrating multiple histone modifications within the same tissue does not enhance age prediction accuracy or robustness. Instead, combining distinct histone marks may dilute or obscure mark-specific aging signals, resulting in diminished predictive performance and reduced sensitivity to age-associated variation.

Given that the correlation patterns between histone mark enrichment and aging exhibit unimodal, bimodal, or even trimodal distributions across different tissues and histone marks (*SI Appendix*, Fig. S4), this variability influenced the number of peaks included in the models. We further investigated whether the number of peaks input into the model, which are peaks potentially related to aging and used for modeling, was significantly associated with the number of features selected by the model and with model performance. Spearman correlation analysis showed no significant association between the number of input peaks and the number of features selected by the model (Spearman’s r = 0.18, *P* = 0.29). However, as expected, a higher number of input peaks was significantly correlated with improved prediction performance (*SI Appendix*, Fig. S8). This finding likely reflects that including more peaks captures richer epigenetic information within the tissue.

To evaluate whether the predictive performance of the clock is constrained by the current training sample size, we performed a sample-size saturation analysis inspired by the MEDIPS framework. Briefly, training samples were randomly divided into two independent subsets, and models were trained using progressively increasing numbers of samples from each subset. Model stability was quantified by the correlation between predictions generated from the two independently trained models. This procedure yields a true saturation curve, reflecting the stability achievable with the available data. To further estimate the theoretical upper bound of model stability, we applied the same analysis to an artificially doubled dataset, generating an estimated saturation curve. Model stability increased rapidly at smaller sample sizes and gradually approached a plateau as more samples were included. The observed saturation correlation reached 0.83 with the available data, while the estimated saturation suggested a modest further increase to approximately 0.89. These results indicate that the current dataset captures most of the predictive structure, and that further increases in sample size are expected to yield diminishing returns.

### Functional Relevance of Model-Selected Histone Peaks.

**Fig. 3.**
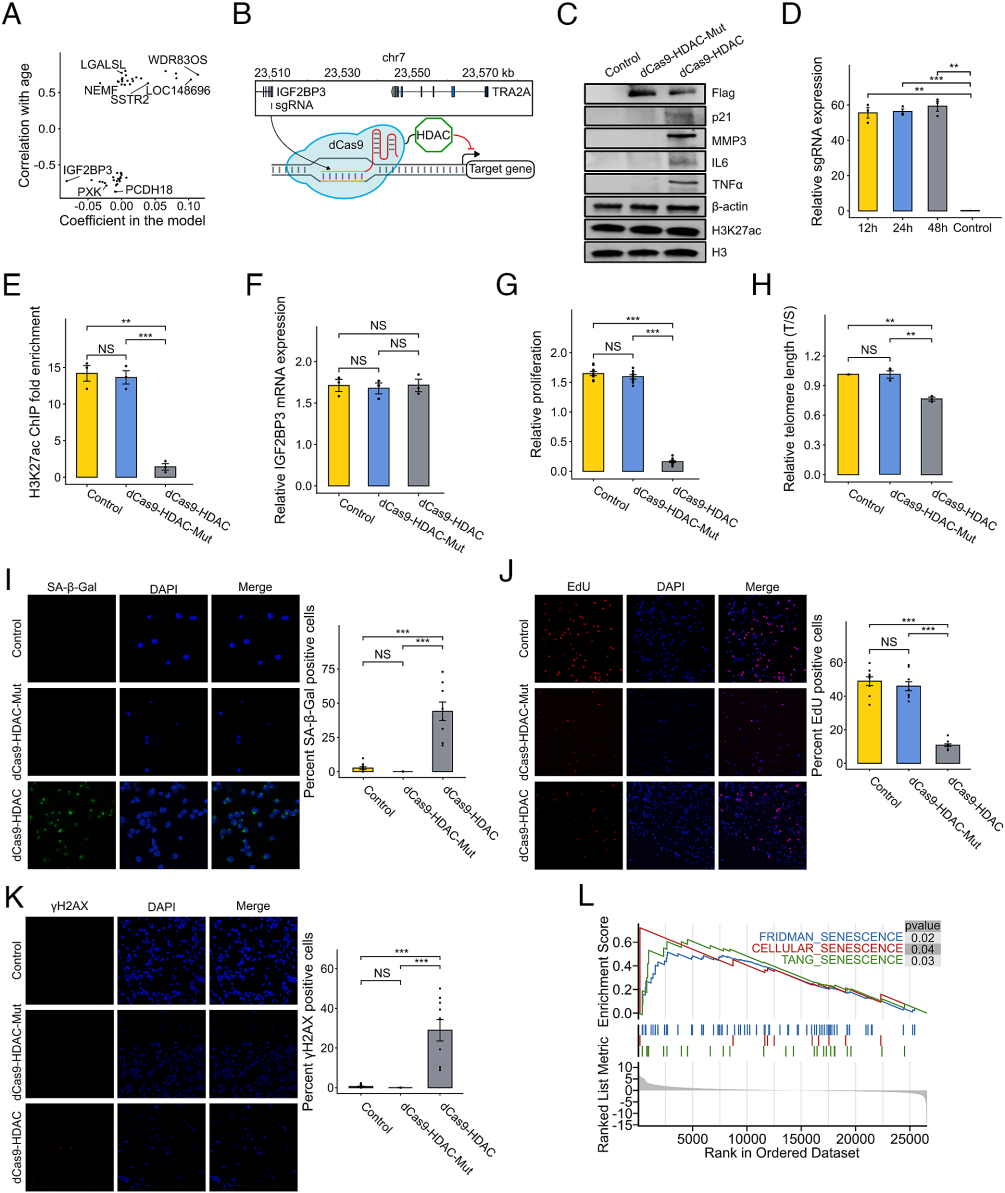
(*A*) Correlation and coefficient of peaks selected by the H3K27ac model in blood. The peaks identified by the model were annotated to their closest genes. The peak at IGF2BP3 exhibits a strong negative correlation with age and had the lowest coefficient. (*B*) Schematic overview of the experimental design for validating the biological role of the age-associated peak near IGF2BP3, targeted using a CRISPR-dCas9-HDAC1 system. (*C*) Western blot of dCas9-HDAC1 complex (Flag) post transfection and senescence-associated markers following target-specific H3K27ac knockdown, with β-actin as a control. Global H3K27ac levels were assessed in parallel, with total H3 as a control, to evaluate whether target-specific dCas9-HDAC1 recruitment induces global changes in H3K27ac. (*D*) Validation of sgRNA expression at 12 h, 24 h, and 48 h posttransfection. (*E*) ChIP-qPCR analysis showing a significant reduction in H3K27ac enrichment at the target locus upon CRISPR-dCas9-HDAC1-mediated knockdown, whereas the mutant dCas9-HDAC1-Mut did not cause a decrease in H3K27ac levels. (*F*) RT-qPCR showing no significant change in IGF2BP3 mRNA expression after H3K27ac suppression. Cell proliferation assessed by CCK-8 assay (*G*) and relative telomere length measured by qPCR (*H*) before and after H3K27ac knockdown. (*I*) Representative immunofluorescence images of SA-β-gal staining in controls and H3K27ac-knockdown cells (*Left*). Nuclei are stained blue; SA-β-gal cells are green. Quantification of SA-β-gal positive cells is shown *Right*). (*J*) EdU incorporation assay assessing proliferation in control and H3K27ac-knockdown cells. Representative immunofluorescence images are shown on the *Left*; quantification of EdU positive cells is on the *Right*. Red: EdU; Blue: DAPI. (*K*) Representative γH2AX immunofluorescence in control and H3K27ac-knockdown cells (*Left*). Quantification of γH2AX-positive cells is shown on the *Right*. Red: γH2AX; Blue: DAPI. (*L*) GSEA of cellular senescence-related pathways following H327ac knockdown. Data are presented as means ± SEM. Mann–Whitney *U* tests or Student’s *t* test were performed for statistical analysis (*P < 0.05, **P < 0.01, ***P < 0.001).

To investigate the functional relevance of loci selected by the histone-based clock, we focused on the blood H3K27ac model, which incorporated 57 nonzero coefficient peaks. While some other histone modifications exhibited clearer separation by age or sex in the PCA or showed slightly better predictive performance, we chose to focus on the blood H3K27ac model for downstream analysis due to its practical and biological relevance. Blood is a clinically accessible tissue, and among blood-based models, H3K27ac displayed a favorable balance between predictive accuracy and robustness. Notably, the H3K27ac clock trained on mixed-sex samples performed substantially better than those trained on male-only or female-only samples (RMSE = 4.94 vs. 19.54 and 15.41, respectively; MAE = 2.54 vs. 17.44 and 11.68). Furthermore, the PCA of H3K27ac profiles showed minimal sex-related variation, suggesting that this mark may capture age-related epigenetic changes in a relatively sex-independent manner. These considerations made the blood H3K27ac model a suitable candidate for functional follow-up. Among these, a peak located near the promoter of IGF2BP3, a regulator within the IGF signaling axis, exhibited a high absolute regression coefficient and strong negative correlation with age (Pearson’s r = −0.7, *P* < 0.01; Fig. 3*A*). Given the known role of IGF2BP3 in modulating senescence and proliferation, we hypothesized that this peak might function as a regulatory element.

To test this, we employed CRISPR dCas9-HDAC1 to target and deacetylate the H3K27ac mark at the selected peak (Fig. 3 *B*–*D*). In addition to the negative control that transfects dCas9-HDAC1 together with a blank sgRNA vector, we also used dCas9-HDAC1-Mut, an H141A mutant of HDAC1, to rule out potential CRISPR off-target effects and any unintended global deacetylation of H3K27ac. Western blot analysis showed that recruitment of either dCas9-HDAC1 or dCas9-HDAC1-Mut did not induce detectable global changes in H3K27ac levels compared with controls (Fig. 3*C*), indicating the absence of widespread epigenetic perturbations. Consistent with this, targeting was validated by ChIP-qPCR, which revealed effective, locus-specific deacetylation following dCas9-HDAC1 recruitment (Fig. 3*E*), whereas the catalytically inactive dCas9-HDAC1-Mut showed no significant deacetylation, confirming the target specificity of this system. Surprisingly, H3K27ac knockdown at this locus did not significantly alter IGF2BP3 mRNA expression (Fig. 3*F*), despite the peak’s proximity to the IGF2BP3 gene. This observation suggested that the regulatory effects of this enhancer might target other genes through long-range chromatin interactions. Nevertheless, this locus-specific H3K27ac knockdown induced profound phenotypic changes associated with cellular senescence. Treated cells exhibited significantly reduced proliferation as measured by CCK-8 assay (Fig. 3*G*) and shortened telomeres (Fig. 3*H*). Western blot analysis revealed increased expression of senescence-associated markers, including p21, IL6, and inflammatory factors (Fig. 3*C*). Cellular senescence was further confirmed by increased SA-β-gal staining in target-specific H3K27ac knockdown cells (Fig. 3*I*), reduced EdU incorporation indicating decreased DNA synthesis (Fig. 3*J*), and elevated γH2AX foci signifying DNA damage accumulation (Fig. 3*K*). Gene set enrichment analysis (GSEA) of RNA-seq data from control and target-specific H3K27ac knockdown cells showed significant enrichment of cellular senescence-related pathways (Fig. 3*L*), further supporting the role of this epigenetic feature in regulating cellular aging.

**Fig. 4.**
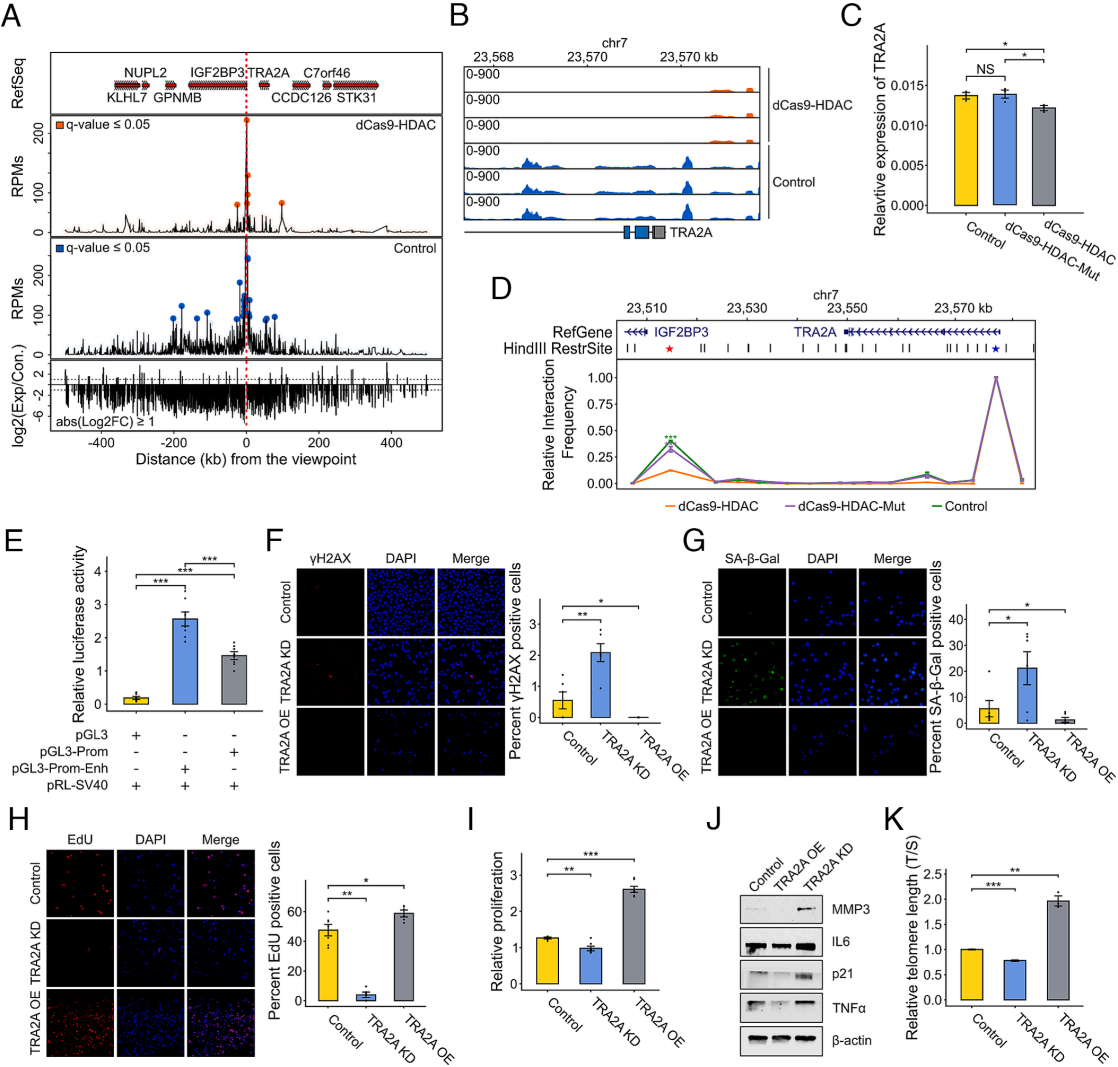
(*A*) Genome browser tracks illustrating 4C-seq data in proximity to the viewpoint. From top to bottom: hg19 gene annotations, read counts per million (RPM) for H3K27ac knockdown cells, RPM for control cells, and log2-transformed fold change of RPM between H3K27ac knockdown and control cells. (*B*) RNA-seq tracks depicting TRA2A expression following H3K27ac knockdown. (*C*) Quantification of relative mRNA expression of TRA2A after H3K27ac knockdown. (*D*) 3C analysis demonstrating the interaction frequency between the TRA2A promoter and a distal enhancer marked by H3K27ac. The *x*-axis represents genomic coordinates, with HindIII restriction sites indicated by black lines; the red star denotes the enhancer location. (*E*) DLR assay showing relative luciferase activity for constructs containing only the enhancer, only the TRA2A promoter, and both the promoter and enhancer. The construct containing both the promoter and enhancer exhibits the highest luciferase activity, while the construct without the promoter shows minimal activity. (*F*) Representative immunofluorescence images of γH2AX in control, TRA2A KD, and TRA2A OE. Quantification of γH2AX-positive cells is shown on the *Right*. Red: γH2AX; Blue: DAPI. (*G*) SA-β-gal staining of senescent cells in control, TRA2A KD, and TRA2A OE cells. Immunofluorescence images are shown on the *Left*, with quantification of SA-β-gal-positive cells on the *Right*. Green: SA-β-gal; Blue: DAPI. (*H*) EdU incorporation assays assessing cell proliferation in control, TRA2A KD, and TRA2A OE cells. Representative immunofluorescence images are shown on the *Left*, with quantification of EdU-positive cells on the *Right*. Red: EdU; Blue: DAPI. (*I*) CCK-8 assay measuring cell proliferation in control, TRA2A KD, and TRA2A OE cells. (*J*) Western blot analysis of senescence-associated markers in control, TRA2A KD, and TRA2A OE cells, with β-actin as an endogenous control. (*K*) Quantification of relative telomere length in control, TRA2A KD, and TRA2A OE cells, as determined by qPCR. Data are presented as means ± SEM. Mann–Whitney *U* tests or Student’s *t* test were performed for statistical analysis (*P < 0.05, **P < 0.01, ***P < 0.001).

To identify downstream targets mediating the observed senescence phenotypes, we first performed 3D genome analysis. Hi-C data revealed five candidate genes located within the same topologically associating domain (TAD) as IGF2BP3. These chromatin interactions were further supported by data from the Enhancer Atlas (*SI Appendix*, Fig. S9). We then performed 4C-seq using the H3K27ac peak region as a viewpoint. This analysis revealed significant interactions with the promoter of TRA2A, which encodes a phosphorylated nuclear RNA-binding protein that regulates pre-mRNA splicing (45), with interaction frequency decreasing following target-specific H3K27ac knockdown (Fig. 4*A*). RNA-seq and RT-qPCR confirmed reduced TRA2A expression after target-specific H3K27ac knockdown (Fig. 4 *B* and *C*), suggesting that TRA2A might be a key target of this regulatory element.

We further validated the enhancer–promoter interaction using 3C analysis, which confirmed physical contact between the H3K27ac peak region and the TRA2A promoter (Fig. 4*D*). Dual luciferase reporter assays demonstrated that the enhancer significantly increased TRA2A promoter activity (Fig. 4*E*), providing additional evidence for its regulatory function.

To determine whether TRA2A mediates the senescence phenotypes induced by H3K27ac knockdown, we performed TRA2A knockdown (KD) and overexpression (OE) experiments (*SI Appendix*, Fig. S10). TRA2A KD recapitulated the senescence phenotypes observed with H3K27ac knockdown, including increased γH2AX foci (Fig. 4*F*), elevated SA-β-gal staining (Fig. 4*G*), reduced EdU incorporation (Fig. 4*H*), and decreased cell proliferation (Fig. 4*I*). Western blot analysis confirmed increased expression of senescence markers in TRA2A KD cells (Fig. 4*J*). Conversely, TRA2A OE partially rescued these phenotypes and extended telomere length (Fig. 4*K*).

These findings establish a mechanistic link between an age-associated histone modification identified by our epigenetic clock and cellular senescence, demonstrating that model-selected features can have causal roles in aging biology. The H3K27ac-TRA2A regulatory axis represents a previously unrecognized epigenetic mechanism controlling cellular aging, highlighting the biological relevance of our histone modification-based clocks.

### Cross-Species Application: Histone-Based Clock in *D. melanogaster*.

A significant limitation in aging research has been the inability to apply conventional epigenetic clocks to species lacking genomic DNA methylation. This constraint is particularly relevant for important model organisms such as *D. melanogaster*, which lacks 5-methylcytosine (5mC) modifications. This gap has hindered comparative studies in evolutionary biology and cross-species aging research, leaving a substantial portion of the animal kingdom without reliable epigenetic aging biomarkers. Histone modifications, being evolutionarily conserved across eukaryotes, offer a promising alternative for developing species-agnostic epigenetic clocks.

**Fig. 5.**
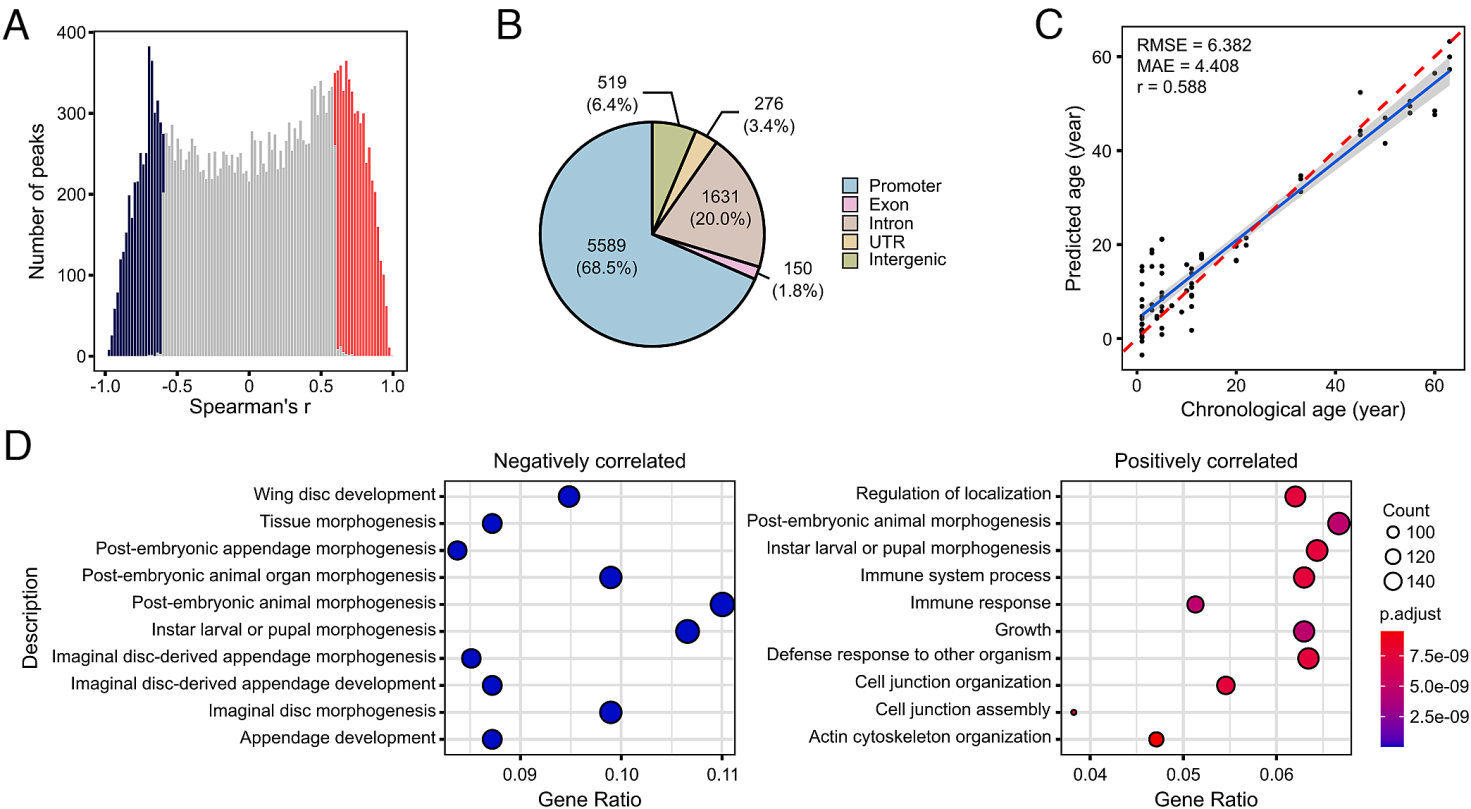
(*A*) Distribution of Spearman’s r between H3K27ac ChIP-seq peaks and age of fruit flies. The *x*-axis represents the correlation coefficient, while the *y*-axis shows the number of peaks. Peaks positively correlated with age (Spearman’s r ≧ 0.5, *P* ≦ 0.05) are highlighted in red, and those negatively correlated (Spearman’s r ≦ −0.5, *P* ≦ 0.05) are highlighted in blue. (*B*) Genomic annotation of age-associated H3K27ac peaks, with significant enrichment in the promoter region (Fisher’s exact test, *P* = 0.0004). (*C*) Age predictions of a clock trained on all H3K27ac ChIP-seq data derived from *Drosophila* and tested on test datasets. RMSE, MAE, and Pearson’s r are indicated. (*D*) GO enrichment of significantly age-correlated peaks with *Left* panel for enrichment of negatively correlated peaks and *Right* panel for positively correlated peaks.

To test the applicability of histone modification-based clocks in nonmammalian systems, we performed H3K27ac ChIP-seq on fruit fly samples across different ages (1, 3, 5, 13, 20, 33, 45, 55, 60, and 63 d posteclosion). Analysis of age-associated H3K27ac peaks revealed 8,566 potentially age-correlated regions (absolute Spearman’s r ≧ 0.5), with 4,703 positively correlated and 3,863 negatively correlated with age (Fig. 5*A*). Genomic annotation showed that these age-associated peaks were distributed across various genomic elements, with enrichment in intronic and promoter regions (Fisher’s exact test, *P* = 0.0004; Fig. 5*B* and Dataset S2).

Using the same modeling approach applied to human data, we constructed a fly H3K27ac-based epigenetic clock. This model demonstrated moderate predictive performance (Pearson’s r = 0.588, RMSE = 6.382, MAE = 4.408; Fig. 5*C*), establishing proof-of-concept for histone-based biological age prediction in a species lacking DNA methylation. While the performance was lower than in human tissues, this likely reflects the smaller sample size and potentially different aging dynamics in flies compared to humans.

Functional annotation of age-associated peaks revealed enrichment in promoters and biological processes related to stress response, immunity, and homeostasis. Peaks with declining H3K27ac were linked to developmental pathways, suggesting a functional shift from developmental regulation to maintenance with age (Fig. 5*D*).

We next assessed whether the nonlinear aging trajectories observed in humans are conserved in fruit flies. In whole-body samples, H3K27ac signal dynamics showed modest changes with age, and the inflection points were not well defined, likely due to the lack of tissue specificity (*SI Appendix*, Fig. S11*A*). Similarly, while the number and length of super enhancers exhibited age-related fluctuations, the trends were subtle and inconsistent (*SI Appendix*, Fig. S11*B*). Typical enhancers showed similarly weak age-dependent patterns (*SI Appendix*, Fig. S11*C*), further suggesting that whole-organism analyses may dilute tissue-specific regulatory dynamics.

These findings demonstrate the cross-species applicability of histone modification-based epigenetic clocks and provide a framework for studying aging in organisms where DNA methylation clocks cannot be applied. The ability to track biological age using histone modifications across evolutionarily diverse species opens avenues for comparative aging research and may help identify conserved mechanisms underlying the aging process.

## Discussion

Aging involves extensive epigenomic remodeling that fundamentally alters gene expression programs and cellular function. Despite significant advances in understanding DNA methylation changes during aging, the tissue-specific dynamics of histone modifications and their regulatory consequences have remained poorly characterized. Our study addresses this knowledge gap through a comprehensive cross-tissue analysis of age-associated histone modification patterns. We demonstrate that histone ChIP-seq signals not only serve as robust predictors of biological age but also reveal previously unrecognized chromatin features of the aging process. By developing and validating histone modification-based epigenetic clocks, we establish a complementary framework to traditional DNA methylation clocks with distinct advantages for certain applications and model systems.

### Histone Modifications and Age: New Insights.

Previous studies have aggregated samples by histone mark while overlooking tissue-specific contexts (35). Such approaches, while informative, risk obscuring biologically meaningful variation that emerges only when both histone mark identity and tissue context are considered simultaneously. Our integrative, multidimensional framework systematically accounts for both axes: histone modification type and tissue specificity, revealing distinct as well as shared aging-associated trajectories across diverse human tissues.

A key finding from our analysis is the tissue-specific directionality of age-associated changes in histone modifications. For instance, we observed that the enhancer-associated H3K27ac increased with age in blood but decreased in the heart and stomach, suggesting that even the same histone modification may play divergent roles depending on tissue context. In contrast, repressive marks such as H3K27me3 and H3K9me3 showed more heterogeneous patterns, potentially reflecting tissue-specific responses to aging-related stress or chromatin compaction.

Notably, we observed pronounced nonlinear trajectories in certain histone marks with inflection points around approximately 40 and 60 y of age across multiple tissues. These ages closely align with previously reported molecular aging transition points, most prominently identified at the protein level in longitudinal multiomics studies, which described coordinated system-wide shifts occurring around ~44 and ~60 y of age (43). The concordance between chromatin-level inflection points and protein aging transitions suggests that mid-life and late-life aging phases may involve discrete reorganization of repressive chromatin states, potentially reflecting upstream epigenetic restructuring that accompanies broader physiological remodeling. Such nonlinear trends may reflect biological thresholds at which chromatin accessibility, transcriptional plasticity, or cellular identity undergo critical shifts that would likely be overlooked in linear modeling approaches. While these observations are correlative, they support a model in which nonlinear changes in histone modifications mark conserved aging transition windows that are detectable across molecular layers. However, given the cross-sectional nature of our histone modification data, future longitudinal epigenomic profiling will be required to directly establish temporal coupling between chromatin remodeling and protein-level aging transitions.

### Histone Modification-Based Clocks: Advantages and Applications.

Building upon these insights, we constructed 36 histone modification-based epigenetic clocks, which exhibited robust predictive accuracy (mean Pearson’s r = 0.91) across multiple tissues and marks. Among these, the blood-derived H3K27ac clock emerged as a particularly powerful model, outperforming several established DNA methylation clocks under matched conditions. This performance is remarkable considering that DNA methylation clocks have undergone extensive optimization over the past decade (9, 16, 18), while our histone-based approach represents a first-generation effort.

A distinctive advantage of our histone-based clocks is their resilience to technical and biological noise. When exposed to artificial Gaussian noise, the histone-based clock maintained stable predictive performance, in contrast to the sharp degradation observed in many methylation-based models. This robustness is likely attributable to the broader, structural nature of histone mark signals, which may be less sensitive to local fluctuations than single CpG methylation values. This characteristic makes histone clocks potentially more suitable for noisy, heterogeneous, or clinically derived datasets where sample quality may vary.

The practical utility of our histone-based clocks was further demonstrated by their ability to detect biological age acceleration in leukemia samples and capture age reversal following therapeutic interventions. These applications highlight the potential of histone-based clocks as biomarkers for disease states and treatment responses, offering a complementary approach to existing clinical tools.

### Super Enhancer Fragmentation and Chromatin Disorganization in Aging.

Another key finding of our study is the phenomenon of super enhancer segmentation, characterized by an increase in super enhancer number alongside a decrease in their average length with age. This structural fragmentation of regulatory domains may reflect a breakdown in enhancer cooperativity, a process that could undermine transcriptional robustness and contribute to cellular heterogeneity during aging. Given the central role of them in maintaining cell identity (46, 47), age-associated super enhancer remodeling may underlie lineage drift and functional decline observed in aging tissues.

This observation is consistent with recent reports of enhancer dynamics in senescent cells and aging stem cell niches, and points to super enhancer architecture as a potential marker or mediator of aging at the chromatin level (47, 48). The progressive disorganization of higher-order chromatin structures during aging may represent a fundamental mechanism through which epigenetic dysregulation contributes to age-related functional decline across tissues.

Moreover, super enhancer segmentation, which is clearly observed in individual tissue datasets, becomes indistinct when all tissues are combined into a single dataset. This highlights the critical importance of preserving tissue specificity in epigenomic analyses, as merging distinct tissue types may obscure biologically meaningful patterns that are otherwise apparent in tissue-specific data.

### From Correlation to Causation: Functional Validation Significance.

Beyond predictive performance, our clocks provide biologically meaningful insights through feature analysis and functional validation. The blood H3K27ac model identified a key peak near the IGF2BP3 locus, a gene implicated in the IGF signaling axis and cellular senescence. Our targeted epigenetic editing experiments demonstrated that inhibition of H3K27ac at this region induced profound changes in senescence phenotypes, including increased SA-β-gal staining, telomere attrition, and upregulation of inflammatory markers.

Through mechanistic studies, we found that this enhancer regulates TRA2A expression through long-range chromatin interactions, establishing a previously unrecognized regulatory circuit in cellular senescence. These findings reveal that certain histone modifications not only correlate with aging but also participate in regulatory networks that influence cellular state, suggesting that epigenetic clock features may have causal, not just correlative, roles in aging biology.

This transition from correlation to causation represents a significant advance in epigenetic clock research, as it begins to bridge the gap between statistical prediction and biological mechanism. By demonstrating the functional relevance of model-selected features, our study provides a framework for identifying potential targets for interventions aimed at modulating biological age.

### Cross-Species Applications and Evolutionary Significance.

Importantly, we extended our framework to *D. melanogaster*, a model organism lacking canonical DNA methylation (31). We successfully constructed a histone modification-based clock using H3K27ac ChIP-seq data, which captured biological age with moderate accuracy. Functional annotation of age-associated peaks revealed a chromatin shift from developmental to stress-response pathways during aging, consistent with evolutionary theories of aging that posit a trade-off between development and maintenance.

This cross-species application demonstrates the potential of histone modifications to serve as universal aging biomarkers, expanding the applicability of epigenetic clocks beyond DNA methylation-dependent systems. Given the evolutionary conservation of histone modifications, such clocks may offer valuable tools for studying aging in nonmammalian systems or species with incomplete methylomes, enabling comparative studies across the evolutionary spectrum.

### Limitations and Future Directions.

While our study establishes histone modifications as powerful biomarkers of biological age, several important limitations must be acknowledged. One notable limitation of our current study is the imbalance in age distribution and sex representation within certain tissues, primarily due to constraints in publicly available datasets. For example, in the stomach tissue, there is a visible gap in age coverage, while the spleen dataset is markedly biased toward a single sex. These imbalances could potentially affect the training and generalizability of the aging prediction models. To assess the impact, we attempted to construct sex-specific models. However, their performance was substantially lower than that of the combined model, likely due to insufficient sample sizes when stratified by sex. These findings suggest that, under the current data limitations, model robustness benefits from combining sexes. Nevertheless, this compromise may obscure potential sex-specific aging patterns. In future studies, increasing the sample size with more balanced age and sex distributions will be crucial to improve model resolution and enable the development of more personalized aging clocks.

Our ChIP-seq analyses were performed on bulk tissue samples, which inevitably average signals across heterogeneous cell populations. This tissue-level resolution may mask cell type–specific aging trajectories that could be particularly informative in complex tissues. Integration with emerging single-cell chromatin profiling technologies will be essential to resolve this heterogeneity and potentially improve model specificity and biological interpretability.

Although our functional validation experiments confirm the causal role of a model-selected H3K27ac peak in senescence regulation, this represents only one of dozens of features identified by our models. Systematic validation of additional age-associated loci is necessary to establish the broader mechanistic relevance of these epigenetic features and their potential as therapeutic targets.

Despite assembling one of the largest cross-tissue histone modification datasets to date, certain tissue-modification combinations remain underrepresented, potentially limiting model robustness in these contexts. Expanded data collection efforts, particularly for tissues showing unique aging patterns, will further strengthen the applicability of histone-based clocks across diverse biological systems.

Future studies should also explore the integration of histone modification data with other epigenetic layers, including DNA methylation, chromatin accessibility, and noncoding RNAs, to develop multiomics clocks that may capture complementary aspects of biological aging. Additionally, longitudinal studies tracking histone modifications over time in the same individuals would provide valuable insights into the stability and predictive power of these epigenetic features.

### Conclusion and Broader Impact.

In summary, our study transforms the paradigm of epigenetic aging clocks by establishing histone modifications as powerful encoders of age-related information with three key advantages: predictive accuracy comparable to gold-standard DNA methylation clocks; remarkable resilience to technical and biological noise; and direct biological interpretability through functional validation. These findings significantly advance our understanding of chromatin dynamics during aging and provide a foundation for developing next-generation aging biomarkers applicable across diverse species and biological contexts.

Histone-based clocks are particularly valuable for model organisms lacking DNA methylation systems and offer complementary insights even in mammals where both epigenetic layers can be assessed. As aging research increasingly focuses on developing interventions to extend health span, these histone modification signatures may serve not only as monitoring tools but potentially as targets for epigenetic reprogramming strategies. By expanding the epigenetic toolkit for quantifying biological age, our work opens avenues for comparative aging research, disease monitoring, and therapeutic development across the evolutionary spectrum.

## Materials and Methods

### Data Collection.

Histone modification ChIP-seq datasets for H3K27ac, H3K27me3, H3K36me3, H3K4me1, H3K4me3, and H3K9me3 in blood, intestine, heart, liver, spleen, and stomach were collected from ENCODE, BLUEPRINT, and Roadmap epigenome projects. We excluded samples if they: 1) were not healthy human tissue or were derived from tissues possibly affected by pathologic conditions, such as tumor-adjacent tissue, 2) lacked information about donor age, 3) were cell lines. Raw sequencing data from ENCODE and Roadmap were deposited in the Sequence Read Archive (SRA) database and were publicly available for download. Access to restrictively accessible data from BLUEPRINT was acquired through request and approval from the Data Access Council (DAC). The accessions for all ChIP-seq data involved in this study were listed in Dataset S1.

### Pipeline of ChIP-seq Analysis.

Quality control was applied to ChIP raw sequencing data using FastQC v0.11.9 (49), followed by trimming adaptors and low-quality reads with Trim Galore! v0.6.7 (50). Clean reads were aligned to the UCSC hg19 reference genome using Bowtie2 v2.4.5 (51). Alignment files were processed to remove unmapped reads, nonprimary alignment, or PCR duplicates using samtools (52). Additional quality control of alignment files included evaluating uniquely mappable reads, nonredundant fraction (NRF), PCR bottleneck coefficients (PBC1 and PBC2), percent of reads in peak (%RiP), normalized strand cross-correlation coefficient (NSC), and relative strand cross-correlation coefficient (RSC) using ChIPQC v1.42.0 (53) and run_spp.R (54). Criteria for discarding low-quality samples are shown in *SI Appendix*, Table S2. Peaks were called using MACS2 v2.2.7.1 (55) with the “-C” flag for input background correction. The “--broad” flag used for broad markers like H3K27me3, H3K36me3, H3K4me1, and H3K9me3. Coverage bigWig tracks were generated from alignment files using the bamCoverage function in deeptools v3.5.1 (56) with scale factors calculated by deeptools multiBamSummary. Peak profiles were generated from the bigWig tracks using deeptools computeMatrix and plotProfile. Peaks were annotated to genomic features (e.g. promoter) and associated genes using ChIPseeker v1.42.1 (57). Human enhancer regions were retrieved from the FANTOM5 project (http://fantom.gsc.riken.jp/5/datafiles/phase2.2/extra/Enhancers/human_permissive_enhancers_phase_1_and_2.bed.gz.)

### Construction and Evaluations of Epigenetic Clocks.

We constructed epigenetic clocks following a multistep protocol. First, a consensus peak set was generated for each tissue and histone modification. Specifically, peaks identified at each age point were filtered for replicate consistency and merged to form subconsensus peak sets per age. These subconsensus peak sets were then combined using the merge function of BEDTools v2.31.1 (58) to obtain a final consensus peak set that captured all potential peak locations, which was used for downstream analysis. Second, raw read counts for each peak in the consensus set were quantified using the TCseq v1.30.0 (59). Read densities were calculated by dividing the raw counts by the peak length (in kilobases), and subsequently normalized by the total number of reads-in-peaks (in millions), as previously described (30). Third, batch effects arising from different laboratories, projects, or instruments were corrected using the ComBat function from the sva v3.54.0 (60). Prior to correction, log2 transformation and background subtraction were applied to the signal matrix. Biological covariates such as age and sex were preserved during batch correction to maintain biological relevance. The effectiveness of batch correction was evaluated using principal component analysis (PCA) before and after adjustment. Finally, elastic net regression models were used to predict biological age from histone modification signals, implemented via the glmnet v4.1-8 (61) in R. Model hyperparameters were optimized using leave-one-group-out cross-validation (LOGO-CV) through the cva.glmnet function supplemented in glmnetUtils v1.1.9 (62) in R. Samples were partitioned into 11 groups based on age stratification, such that each group spanned a similar age range. In each LOGO-CV iteration, all samples from one group were held out as the test set, and the model was trained on the remaining groups. Feature values were standardized prior to model training (30). Model performance was evaluated using RMSE, MAE, and Pearson’s r.

To compare and evaluate the performance of our epigenetic clocks with other clocks derived from various omics data, we included the ATAC-clock (30), histone clocks developed by Camillo (35), and DNAm clocks obtained from ComputAgeBench (44). Clock performance was assessed by calculating the quantile rank of the RMSE of our clock relative to the RMSE distribution of 100 predictions for comparison. To assess the stability of the clocks, we introduced random Gaussian noise with SD ranging from 0 to 1.5, increasing in increments of 0.3. To assess the prediction performance of the models, we incorporated datasets from leukemia patients and drug-treated cell lines before and after treatment from NCBI BioProject: PRJNA292119 (63), PRJNA339128 (64), PRJNA789270 (65), PRJNA754174 (66), PRJNA892939 (67).

To evaluate how model prediction accuracy scales with sample size, we performed a sample-size saturation analysis inspired by the saturation framework implemented in the MEDIPS v1.58.0 (68). Briefly, training samples were randomly split into two independent subsets, and models were trained using progressively increasing numbers of samples from each subset. Model stability was quantified by comparing predictions generated by the two independently trained models on the same held-out test dataset. Specifically, for each sample size, Pearson’s r was computed between the predicted ages from the two models. To reduce stochastic variation introduced by random subsampling, the analysis was repeated multiple times for each sample size, and performance metrics were averaged across repetitions. In addition to the observed saturation curve derived from the original dataset, a MEDIPS-style estimated saturation analysis was performed by applying the same procedure to an artificially doubled dataset, allowing us to approximate performance convergence at larger effective sample sizes without introducing additional model assumptions. All analysis code is available in the Data Availability.

Additional experimental procedures, including cell culture, ChIP experiments, CRISPR-dCas9 perturbation, RNA-seq, chromatin interaction assays (Hi-C, 4C, and 3C), luciferase reporter assays, and functional analyses, are described in *SI Appendix*, Materials and Methods.

## Supplementary Material

Appendix 01 (PDF)

Dataset S01 (XLSX)

Dataset S02 (XLSX)

Dataset S03 (TXT)

Dataset S04 (PDF)

## Data Availability

The codes used in this study are deposited on GitHub: https://github.com/zhixinniu/HistoneAgingClock (69). The raw sequencing and processed data are available in the Gene Expression Omnibus under accession number GSE296812 (70). Other data supporting the findings of this study are available via Zenodo at https://doi.org/10.5281/zenodo.18091518 (71). Previously published data were used for this work [Dataset S1, PRJNA292119 (63), PRJNA339128 (64), PRJNA789270 (65), PRJNA754174 (66), and PRJNA892939 (67)]. Other data are included in the article and/or supporting information.
