# Supplementary material for: Histone modification clocks for robust cross-species biological age prediction and elucidating senescence regulation
Source: Proc Natl Acad Sci U S A. 2026 Mar 10;123(11):e2533687123. doi: 10.1073/pnas.2533687123 (PMC12993953; doi:10.1073/pnas.2533687123)
Supplement: Supplementary file 1 — Appendix 01 (PDF) [file pnas.2533687123.sapp.pdf]

PNAS

Supporting Information for
Histone Modification Clocks for Robust Cross-Species Biological Age Prediction and Elucidating Senescence Regulation

Zhixin Niu, Chang Liu, Yurong Fan, Lei Gu

Lei Gu
Email: lei.gu@mpi-bn.mpg.de

This PDF file includes:

Supporting text
Figures S1 to S11
Tables S1 to S5

Other supporting materials for this manuscript include the following:

Datasets S1 to S4

Supporting Information Text

Materials and Methods

Cell Culture. HL-60 cell line, isolated from the peripheral blood of an acute promyelocytic leukemia patient, was used for *in vitro* experiments. HL-60 cells were maintained in RPMI-1640 medium (Sigma-Aldrich) supplemented with 10% FBS (Gibco) and 100 U/mL Pen/Strep (Gibco) under standard conditions (37 °C, 5% CO_2). Cells were passed into new medium every 2 to 3 d.

Drosophila Stocks and Husbandry. *w*1118 fly line was obtained from the Bloomington Drosophila Stock Center. The flies were maintained in a humidified 25 °C room with 12 h:12 h light: dark cycles. Flies were developed on 4% brewer's yeast, 3% dextrose, 2% potato starch, and 0.7% agar medium supplemented with 5 mL of propionic acid and 12 mL of 10% nipagin in 95% ethanol to prevent microbial growth.

Chromatin Immunoprecipitation (ChIP) and Sequencing. For ChIP experiments with the cell line, all procedures followed the manufacturer's instructions for the Pierce Agarose ChIP Kit (Pierce). For fruit fly ChIP-seq, adult flies at 3, 5, 13, 20, 33, 45, 55, 60, and 63 d posteclosion were collected. We modified previously described protocols to isolate fly nucleus (1, 2). Briefly, flies were collected into a Dounce tissue grinder (Wheaton) prefilled with the Homogenization buffer [250 mM sucrose, 25 mM KCl, 5 mM $MgCl_2$, 0.1% Triton X-100, 0.1 mM DTT, 10 mM Tris pH 8.0, and 1× Proteinase inhibitor (Sigma-Aldrich)] and homogenized with >20 strokes using a loose pestle followed by >40 strokes with a tight pestle. The homogenate was filtered through two layers of Miracloth (Millipore) and centrifuged at 1,000× g for 10 min at 4 °C. The cytosolic layer was discarded, and the pellet was resuspended in PBS. The nuclear solution was then transferred into a new homogenizer and disrupted with 10 strokes using a loose pestle. The solution was again filtered with Miracloth, the filtrate was now ready for downstream ChIP experiments. The fly ChIP-ed DNA were used to generate sequencing libraries and sequenced on the Novaseq X platform (PE150) by the Biomarker Technologies (BMK) GmbH, Germany.

Immunoblotting. The cells were lysed in RIPA buffer containing protease/phosphatase inhibitor cocktails (Sigma-Aldrich) and PMSF (Roche). The extracted proteins were denatured at 98 °C for 5 min in 2× Laemmli buffer (Bio-Rad) before being loaded onto Any KD Mini-PROTEAN Precast gels (Bio-Rad). Protein bands were transferred to a PVDF membrane using the Trans-blot Turbo Transfer pack (Bio-Rad). After blocking the membrane with EveryBlot Blocking Buffer (Bio-Rad) for 30 min, it was washed with TBST for 5 min. The membrane was then incubated overnight at 4 °C with primary antibodies diluted in 0.5% BSA/TBST. The antibodies indicated in *SI Appendix*, Table S3. Following three washes with TBST, the membrane was incubated for 1 h with Anti-rabbit HRP-conjugated antibody (Cell Signaling Technology) diluted in TBST at a 1:5,000 dilution. The membrane was washed three times with TBST, and Clarity Western ECL substrate (Bio-Rad) was applied for 5 min. The protein bands were visualized using a ChemiDoc imaging system (Bio-Rad).

RNA Isolation and Quantitative Real-Time PCR Analysis. Cells were collected, and total RNA was isolated with the RNeasy Mini Kit (Qiagen). cDNA synthesis was conducted with the LunaScript RT SuperMix Kit (NEB). Luna Universal qPCR Master Mix was used for real-time qPCR on a CFX Opus 96 system (Bio-Rad). The data were analyzed with CFX Maestro software v2.2.

Target-specific Knockdown of H3K27ac. Target-specific knockdown of H3K27ac was performed using the CRISPR-dCas9 system. The plasmid expressing sgRNA which specifically binds to the selected H3K27ac peak was constructed by cloning a synthesized double-strand DNA corresponding to the sgRNA sequence into the pCR-Blunt II-TOPO vector (Invitrogen) (*SI Appendix*, Table S5). The p-dCas9-HDAC1, a gift from Stephan Beck (UCL Cancer Institute, University College London, London, United Kingdom; Addgene #104409), was used for transient expression of dCas9-HDAC1 fusion protein. Both plasmids were co-transfected into HL-60 cell using the Amaxa Cell Line Nucleofector Kit V (Lonza). As a negative control, cells were transfected with dCas9-HDAC1 and an empty pCR-Blunt II-TOPO vector lacking sgRNA. A vector encoding the HDAC1 H141A mutant (p-dCas9-HDAC1-H141A) was a gift from Stephan Beck (UCL Cancer Institute, University College London, London, United Kingdom; Addgene #104417) and was transfected along with the sgRNA vector to rule out off-target effects and potential alterations in global H3K27ac levels. Two days post-transfection, cells were harvested to validate the expression of sgRNA and dCas9-HDAC1 proteins using RT-qPCR and western blot.

H3K27ac ChIP-qPCR was conducted to verify the knockdown of H3K27ac at the target region. Antibodies and primers are provided in *SI Appendix*, Table S3 and S4.

Bulk RNA-seq and Analysis. RNA was isolated from the control and H3K27ac KD cells using the RNeasy Mini Kit (Qiagen). RNA was further processed for sequencing by the Biomarker Technologies (BMK) GmbH, Germany (non-stranded cDNA libraries; 250 bp paired-end run with a depth of reads per library). Bulk transcriptomes were aligned using Hisat2 v2.2.1 (3) with hg19 reference and quantified using StringTie v2.2.1 (4). Differential expressed genes were determined as *P* value less than 0.05 and the absolute of fold change greater than one using DESeq2 v1.46.0 (5). GSEA was performed using the clusterProfiler package v4.14.6 (6) using the gene sets related to senescence from MsigDB v2024.1.Hs. Gene sets were enriched if the *P* value was less than 0.05.

Immunofluorescence and Microscopy. Suspension cells were seeded on poly-L-lysine coated chamber slides (ibidi) and allowed to attach for 30 min at 37 °C. Then cells were washed twice with PBS and fixed with 4% paraformaldehyde in PBS for 30 min at room temperature, followed by three washes with PBS. Subsequently, cells were allowed for permeabilization with 0.5% Triton X-100 in PBS for 20 min. Following three additional washes with PBS, cells were blocked with 5% BSA in PBS for 1 h at room temperature. The blocking buffer was then removed, and cells were incubated overnight at 4 °C with rabbit Anti-γH2AX antibody (CST) diluted at 1:250 in blocking buffer. The next day, cells were washed three times with PBS for 5 min each. Goat Anti-rabbit DyLight 594 (Invitrogen) was diluted 1:500 in blocking buffer and applied to each well for 1 h at room temperature, protected from light. The secondary antibody was then removed, and cells were washed three times with PBS. Mounting medium with DAPI was applied on the slide. Confocal microscopy images were captured using LAS X software 3.7.5 (Leica) with a SP8 X White Light Laser Confocal Microscope (Leica) and 20× objective.

CCK-8 Assay and Click-iT Edu-Incorporation Assay. Cell proliferation was assessed with CCK-8 assays. Cells were seeded in 96-well plate and incubated with 100 µL of medium supplemented with 10 µL CCK-8 (Sigma-Aldrich) for 2 h at 37 °C. The absorbance was measured at 450 nm to determine cell proliferation. The Click-iT EdU reaction was performed according to the instruction of the Click-iT imaging kit (Invitrogen). For suspension cells, slides should be coated with poly-L-lysine to promote cell adhesion. Images per condition were acquired using a 20× objective. The number of EdU positive cells and DAPI positive cells was determined using ImageJ (7).

SA-β-Gal Assay. The SA-β-Gal assay was conducted following the manufacturer's protocol of the CellEvent Senescence Green Detection Kit (Invitrogen). Cells were washed twice with PBS, then fixed for 15 min with 4% paraformaldehyde in PBS at room temperature, washed with 1% BSA in PBS three times and then incubated for 2 h at 37 °C without CO_2 in working solution. The samples were washed three times with PBS before imaging by microscopy.

Telomere Length. Relative telomere length was determined by qPCR based on a previously published protocol (8, 9). Genomic DNA was isolated using the DNeasy Blood & Tissue Kit (Qiagen) according to the manufacturer's instruction. The genomic DNA was then used as template to conduct qPCR using the Luna Universal qPCR Master Mix with the primers indicated in *SI Appendix*, Table S4. The relative telomere length for each sample was expressed in T/S (telomere to single copy gene) ratio and was calculated: $T/S = 2^{-(C_t^{Telo} - C_t^{SCG})}$ where C_t^{Telo} and C_t^{SCG} are the C_t value for the telomere sequence and single copy gene respectively.

Hi-C Analysis. Hi-C datasets for HL-60 cell line can be found in GSE93997. The raw sequencing data were processed using runHiC v0.9.0 pipeline (10). The final mcool files were divided into cool file with a resolution of 25 Kb using HiCExplorer hicConvertFormat v3.7.2 (11). The cool files were then normalized and corrected using hicNormalize and hicCorrectMatrix. The topologically associating domains were identified by hicFindTADs. The interaction between TRA2A promoter and enhancers were checked from Capture Hi-C data deposited on EnhancerAtlas 2.0 (12). Visualization of Hi-C and Capture Hi-C data were conducted using the pyGenomeTracks v2.7 (13).

4C-seq. 4C-seq was performed to investigate all potential interactions associated with the selected H3K27ac peak following the previous protocols (14, 15). In brief, cells were fixed in 2% formaldehyde isolation buffer and quenched with glycine to a final concentration of 0.13 M. After fixation, the cells were washed once, resuspended in cold cell lysis buffer (50 mM Tris-HCl, pH

7.5, 0.5% NP-40, 1% Triton X-100, 150 mM NaCl, 5 mM EDTA, 1× protease inhibitor cocktail), and incubated on ice for 30 min. The lysed samples were centrifuged to remove the supernatant, and the pellets were resuspended in 450 μL of 1.2× reaction buffer for the first restriction enzyme digestion. SDS was added to a final concentration of 0.3%, and the samples were incubated at 37 °C for 1 h. Triton X-100 was then added to a final concentration of 2%, and the samples were incubated for another hour at 37 °C. The chromatin was digested with DpnII for 4 h at 37 °C, followed by the addition of a second aliquot of DpnII and overnight digestion under the same conditions. After enzyme inactivation by heat, the digested samples were transferred to 50 mL Falcon tubes and mixed with 700 μL of 10× ligation buffer (660 mM Tris-HCl, pH 7.5, 50 mM $MgCl_2$, 50 mM DTT, 10 mM ATP), 7 mL ddH_2O, and T4 DNA ligase. Ligation was performed overnight at room temperature. The ligated samples were reverse crosslinked at 65 °C overnight with Proteinase K, followed by RNase A treatment at 37 °C for 30 min. The DNA was purified using phenol/chloroform extraction. For the second round of restriction digestion, 450 μL of the 3C template was mixed with 50 μL of 1.2× reaction buffer and NlaIII enzyme, and the mixture was incubated overnight at 37 °C. The digested samples were then ligated as described above and purified using phenol/chloroform extraction. The purified DNA was amplified by PCR using primers listed in the *SI Appendix*, Table S4 and the Expand Long Template PCR system (Roche). Amplified 4C-PCR products were purified with the QIAquick PCR Purification Kit (Qiagen). The final products were sequenced by Biomarker Technologies (BMK) GmbH, Germany using the Illumina platform, and the 4C-seq data were analyzed with the r3Cseq v1.52.0 (16).

3C. The 3C assay was performed as previously described (17, 18). The genomic region between the TRA2A promoter and the selected peak was divided into 22 segments by HindIII restriction endonuclease. The fragment containing the TRA2A promoter was designated as the "anchor." Primers for the anchor and other regions are listed in *SI Appendix*, Table S4. qPCR was used to quantify the interactions between the anchor and the other genomic regions.

Dual Luciferase Reporter Assay. Three luciferase plasmids were constructed: (i) pGL3-Basic plasmid, which was a gift from Debrya Groskreutz (Promega Corp., USA; Addgene #212936), (ii) pGL3-Basic-prom plasmid, in which the TRA2A promoter was inserted upstream of the luciferase between the NheI and HindIII sites, and (iii) pGL3-Basic-prom-enh, which contained both the TRA2A promoter and the selected H3K27ac peak inserted between the SalI and BamHI sites. A Renilla luciferase expression plasmid (pRL-SV40) was a gift from Ron Prywes (Columbia University Irving Medical Center, NY, USA; Addgene #27163) and was co-transfected with the pGL3 plasmids to serve as an internal control for normalization. After 24 h of transfection, the cells were washed and harvested in passive lysis buffer (Promega). Firefly and Renilla luciferase activities were measured using a Dual-Luciferase Reporter Assay System (Promega) following the supplier's protocol. Fluorescence emission was detected using a microplate reader (Tecan), and results are presented as the ratio of firefly to Renilla luciferase activity. Primers used in this assay are listed in *SI Appendix*, Table S4.

KD and OE of TRA2A. KD of TRA2A was achieved by transfecting cells with siRNAs. Two siRNAs targeting TRA2A were purchased from Sigma-Aldrich and ThermoFisher (*SI Appendix*, Table S5). A total of 30 pmol of each siRNA was transfected into the cells. The OE plasmid of TRA2A was constructed as follows. Total RNA was isolated from cells, and cDNA was synthesized via reverse transcription. Primers were designed to amplify the TRA2A CDS by PCR using Phusion High-Fidelity PCR Master Mix (NEB). The amplified sequence was then ligated into the pcDNA3.4 vector (Invitrogen). Following both KD and OE of TRA2A, expression levels were quantified by RT-qPCR using specific primers listed in *SI Appendix*, Table S4.

Statistical Analysis. Statistical analysis was carried out in R. Correlation between peak signal value and age was analyzed using Spearman's correlation. Enrichment of genomic features (e.g., promoters and enhancers) among age-associated peaks was assessed using Fisher's exact test by comparing the number of promoter-overlapping and non-promoter-overlapping peaks between the age-associated group and the background set (all tested peaks) in a 2 × 2 contingency table. Correlation between predicted age and actual age was calculated by Pearson's correlation. Statistical comparisons between each pair of groups were conducted using the Mann-Whitney U test or Student's t test, depending on whether the data conformed to a normal distribution, as assessed by the Shapiro-Wilk test. Significant enrichment test of histone modification peaks in

promoter region was completed by Fisher's exact test. NS $P > 0.05$, *$P < 0.05$, **$P < 0.01$, ***$P < 0.001$.

Figures

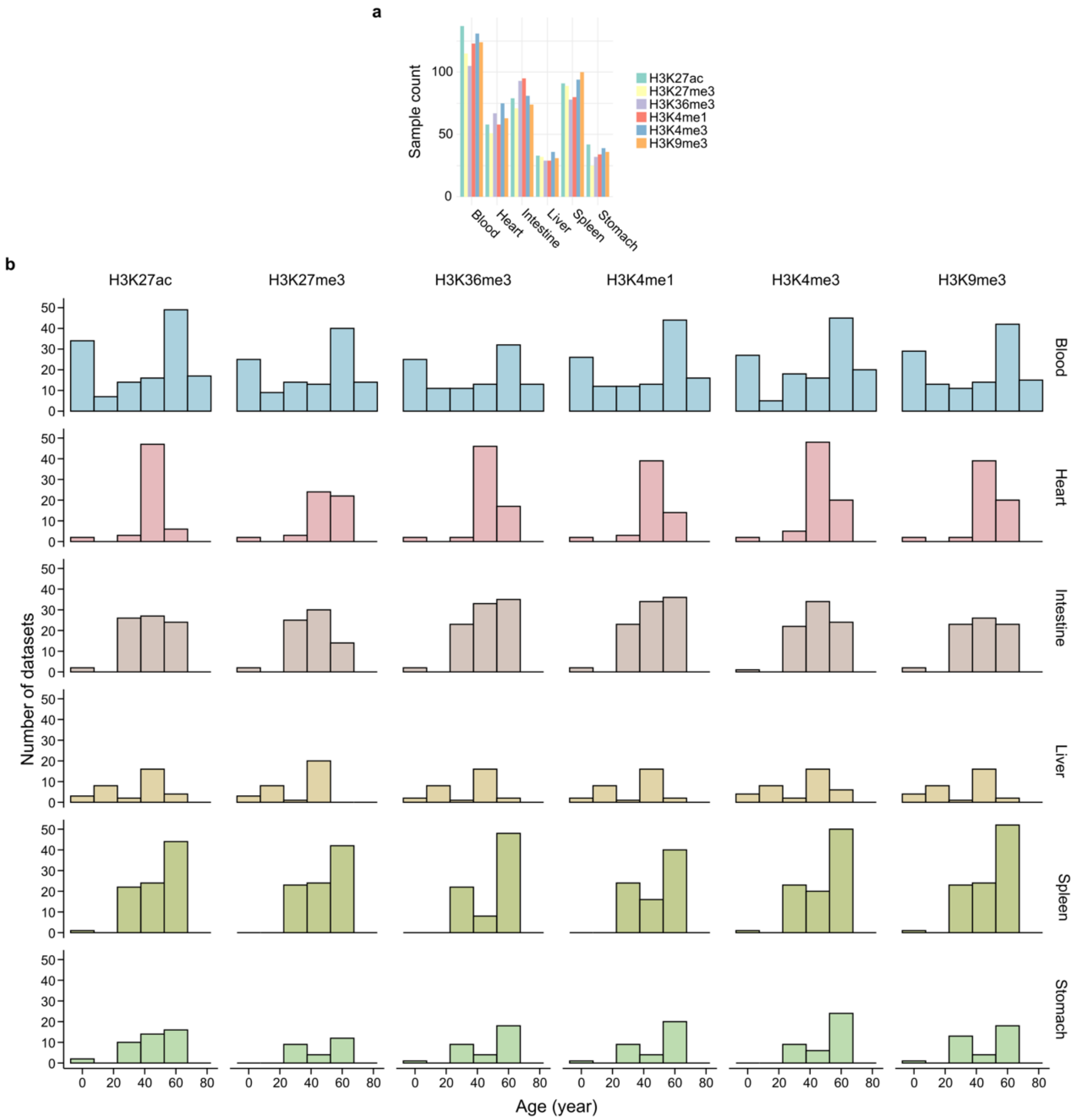

Figure S1. (**a**) Summary of the number of ChIP-seq datasets across different tissues and histone marks. (**b**) Distribution of dataset counts for each histone modification and tissue across age groups.

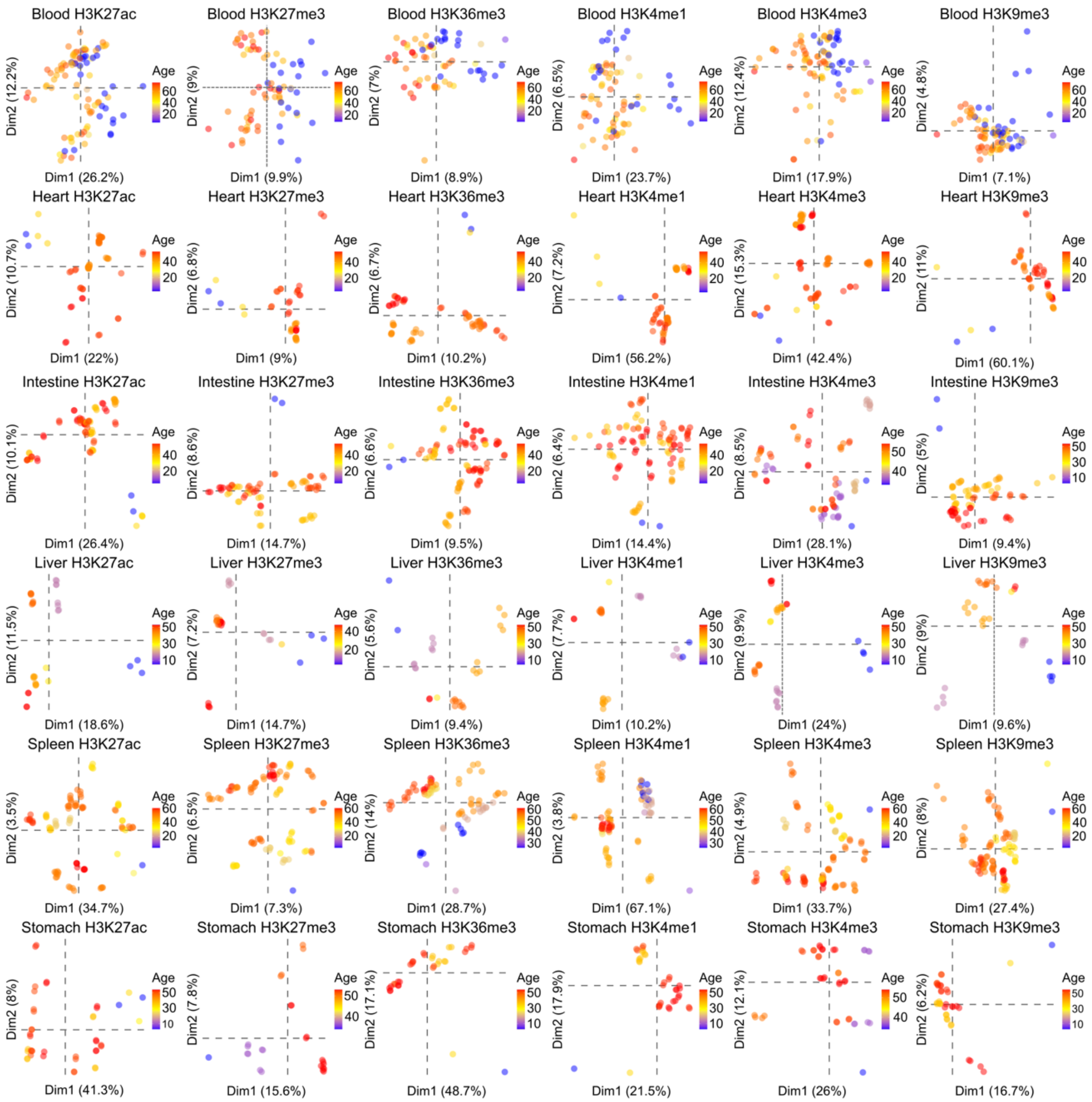

Figure S2. Reduced-dimension PCA plots for each tissue and histone mark, with individual datasets colored by chronological age.

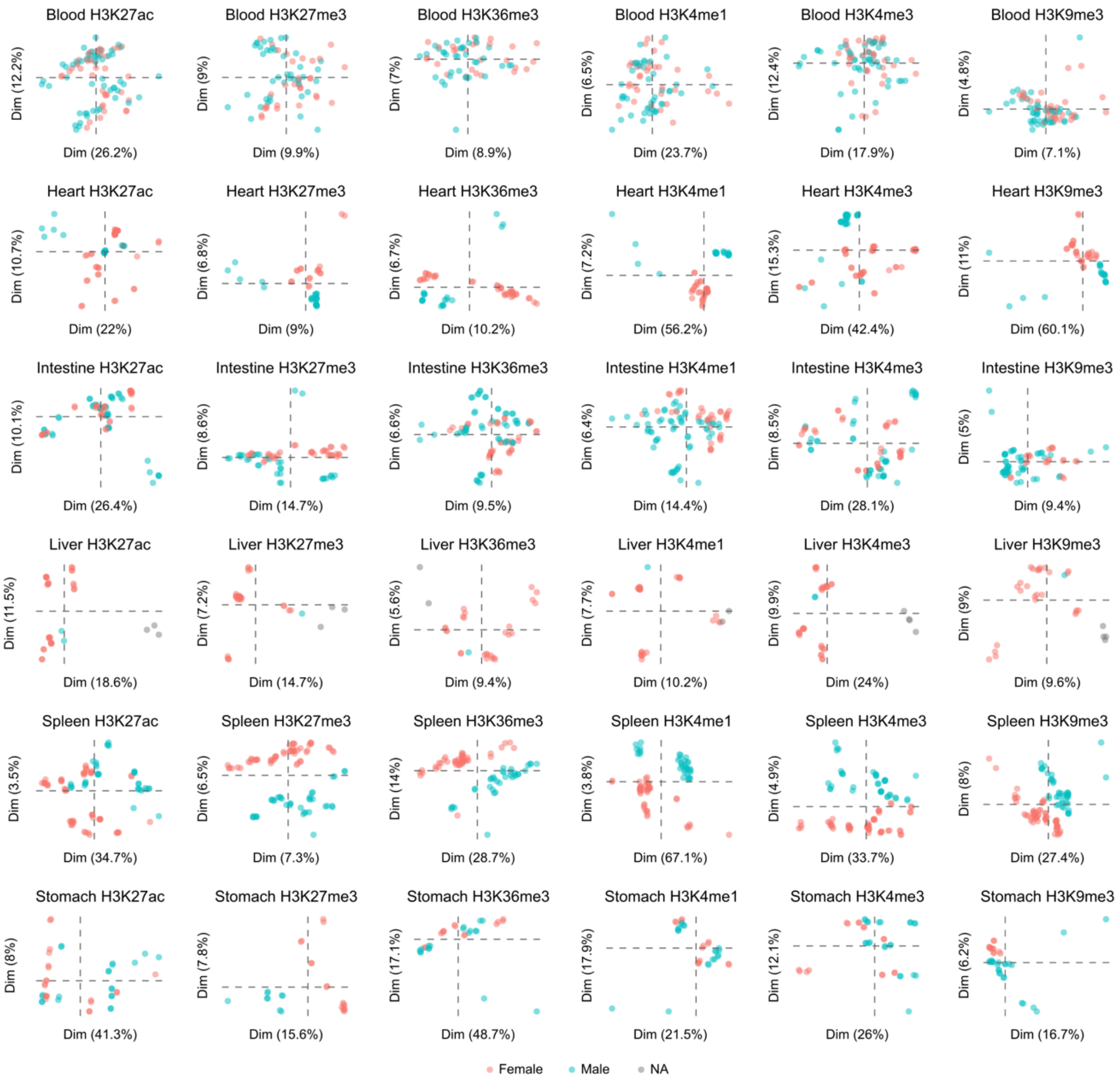

Figure S3. Sex-based distribution of histone modification profiles. PCA plots for each tissue and histone mark, with individual datasets colored by sex.

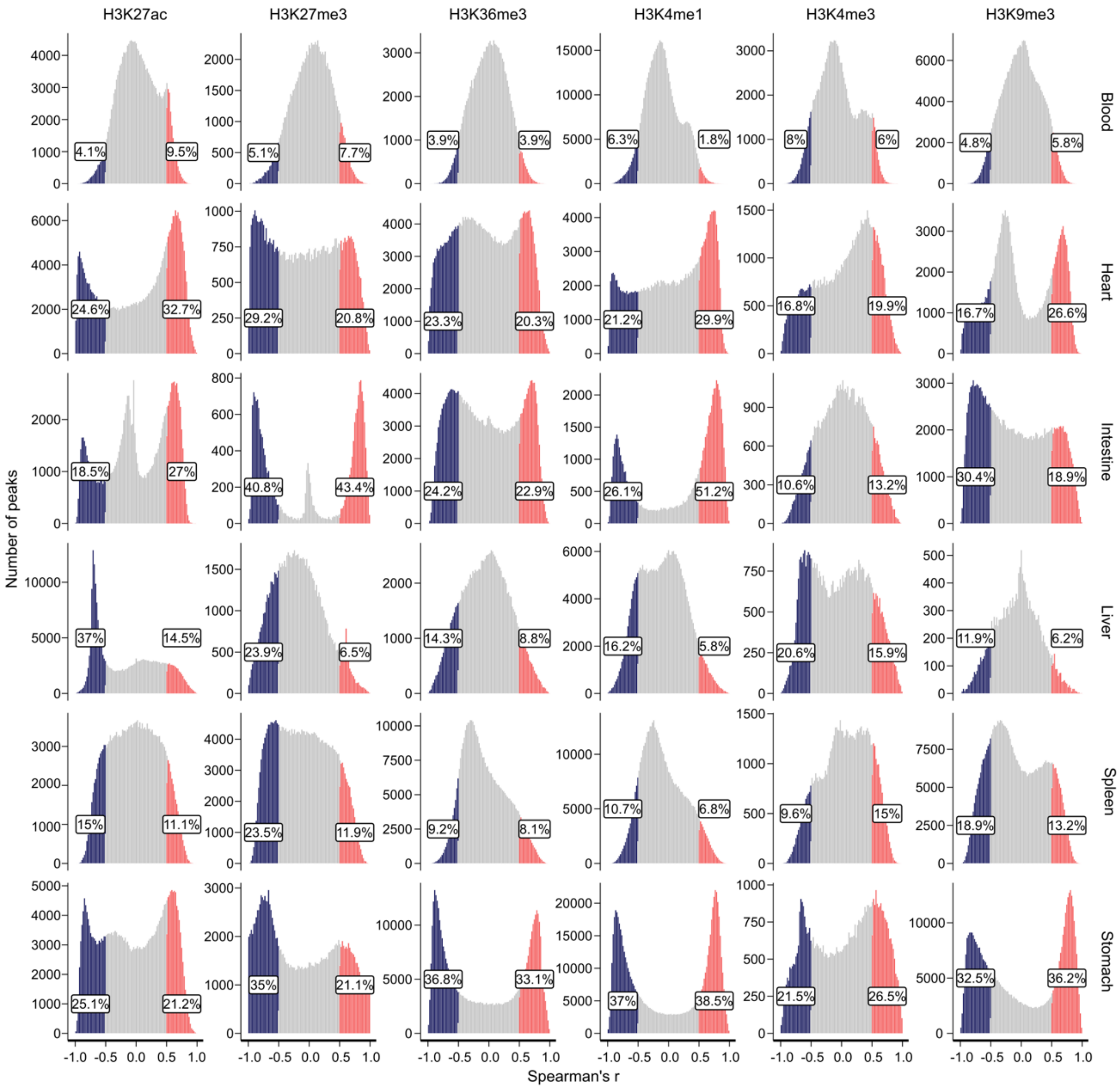

Figure S4. Histogram showing the distribution of Spearman’s r between peak signal intensity and chronological age for each tissue and histone mark. Peaks with significant positive correlations (Spearman’s r ≧ 0.5, *P* ≦ 0.05) are shown in red, and those with significant negative correlations (Spearman’s r ≦ -0.5, *P* ≦ 0.05) are shown in blue. The proportion of positively and negatively correlated peaks relative to the total number of peaks is indicated in each plot.

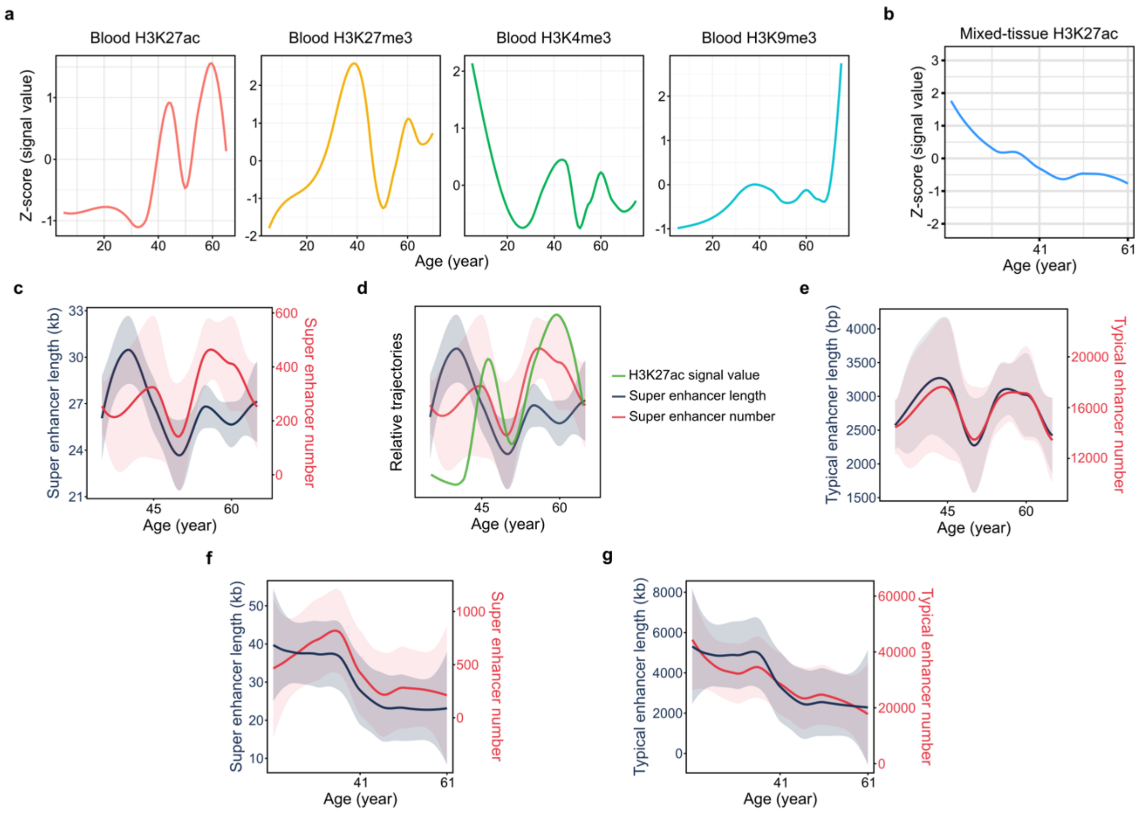

Figure S5. (**a**) Loess regression illustrating age-associated changes in normalized peak signal intensity for H3K27ac, H3K27me3, H3K4me3, and H3K9me3 in blood samples, exhibiting elevated enrichment observed in individuals around 45 and 60 years of age. (**b**) Signal value of H3K27ac peaks across age after combining all tissue types. (**c**) Super enhancer segmentation in blood samples reveal age-associated changes: younger individuals display fewer but longer super enhancers, whereas older individuals show a higher number of super enhancers with reduced average length. (**d**) A merged plot combining the blood H3K27ac trajectories shown in panels (**a**) and (**c**), with H3K27ac signal intensity, super-enhancer length, and super-enhancer number aligned on a shared age axis. (**e**) Typical enhancers show age-associated changes, with both the number and length of typical enhancers varying synchronously across age groups. (**f**) Age-associated changes in super enhancer length and number across combined tissue datasets. (**g**) Trends in typical enhancer length and number across datasets combining multiple tissues.

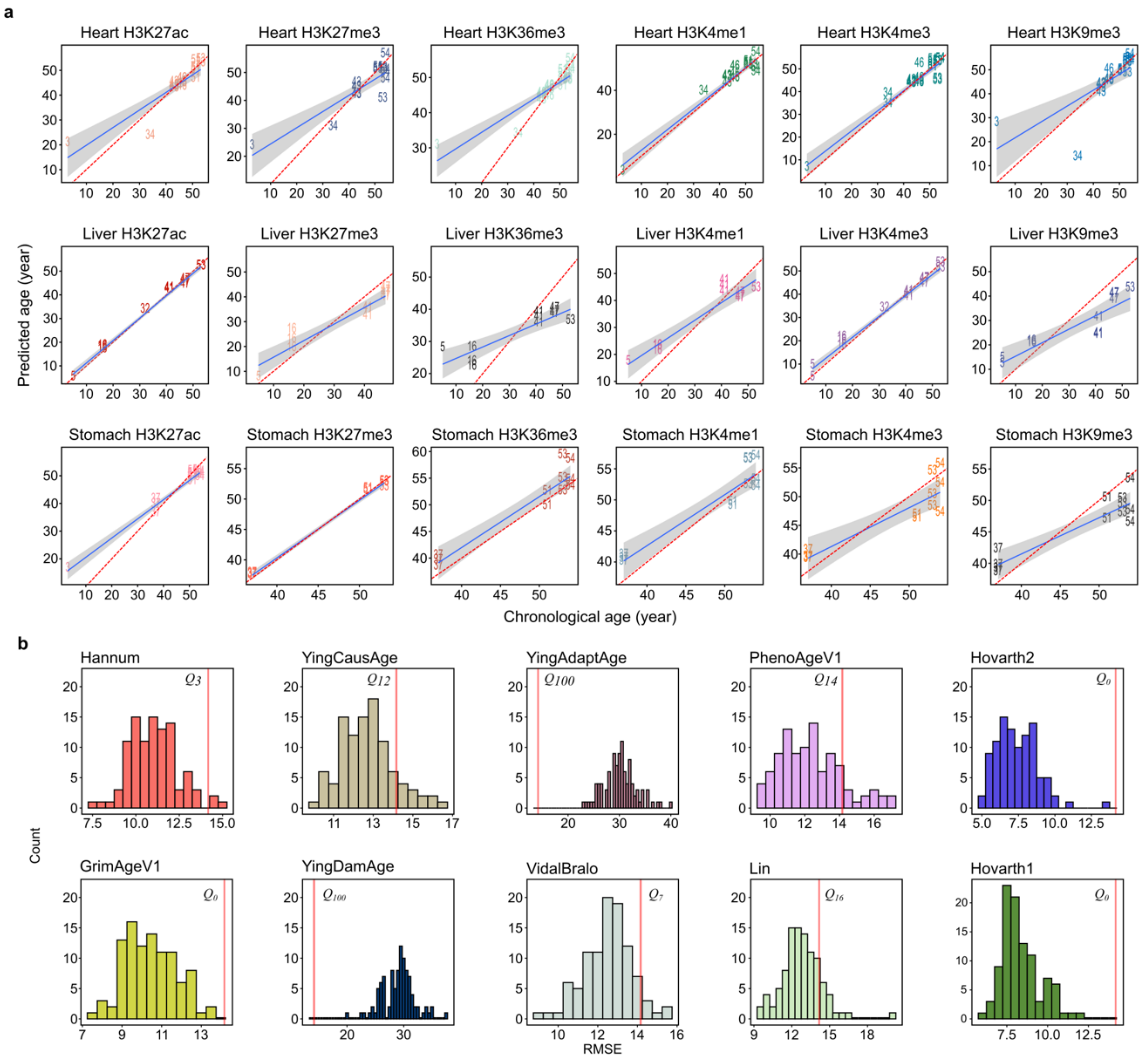

Figure S6. (**a**) Scatter plots comparing predicted age to actual chronological age across three tissues, heart, liver, and stomach, for each histone mark. The red dashed line represents the ideal fit, and the colored solid line denotes the fitted regression line with shaded 95% confidence intervals. RMSE, MAE, and Pearson's r values are reported in Table S1. (**b**) Histogram showing the distribution of RMSE values from age predictors trained on 100 random subsamples from the ComputAgeBench DNA methylation dataset. The vertical-colored line represents the RMSE of the predictor trained using histone modification data.

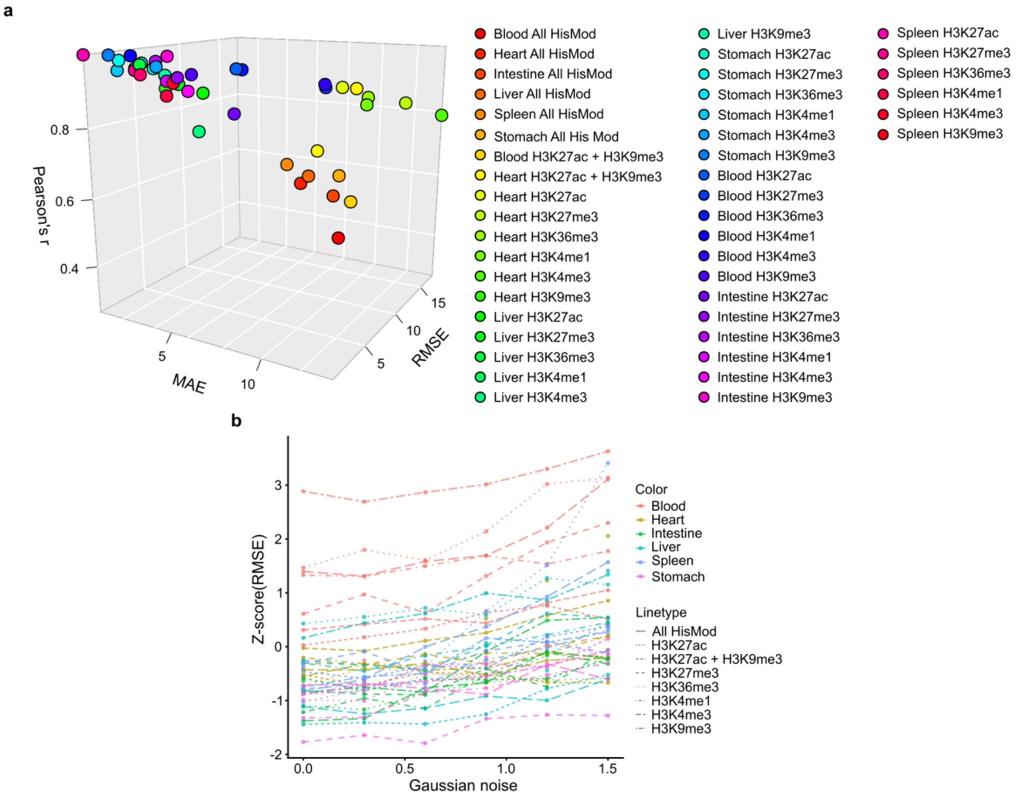

Figure S7. (**a**) Three-dimensional scatter plot summarizing model performance across RMSE, MAE, and Pearson's r. Each point represents an individual aging clock. Shown are tissue-specific single histone mark clocks and multi-histone modification clocks constructed for a single tissue. (**b**) Robustness of aging clock predictions under increasing levels of artificially added Gaussian noise. Noise was introduced to the model input features, and prediction performance was evaluated using the same cross-validation framework as in (**a**). Integrated models were compared with tissue-specific single-mark clocks to assess sensitivity to noise-induced perturbations.

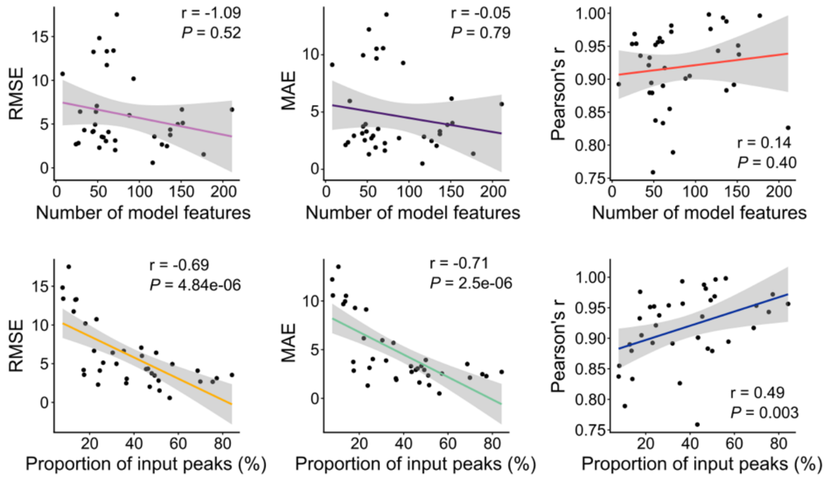

Figure S8. Scatter plots illustrating the correlation between peak numbers and model performance. The upper panels depict the correlation between the number of features selected by the aging clocks and three performance metrics: RMSE, MAE, and Pearson's r. The lower panels show the correlation between the total number of input peaks used for model training and the same performance metrics. Spearman's r and the corresponding *P* value are indicated in each panel.

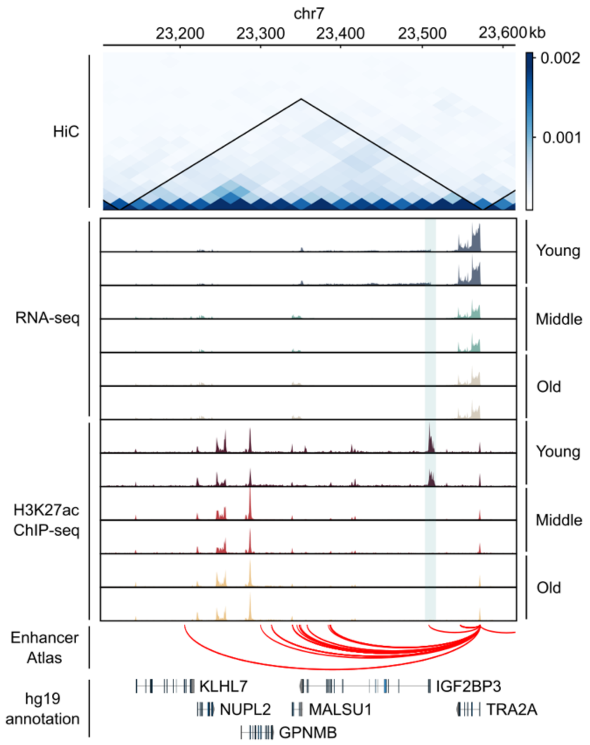

Figure S9. An age-associated H3K27ac peak located near the IGF2BP3 gene locus. From top to bottom: the corresponding TAD, RNA expression levels, H3K27ac signal tracks, enhancer-promoter interactions involving TRA2A, and the hg19 gene annotation.

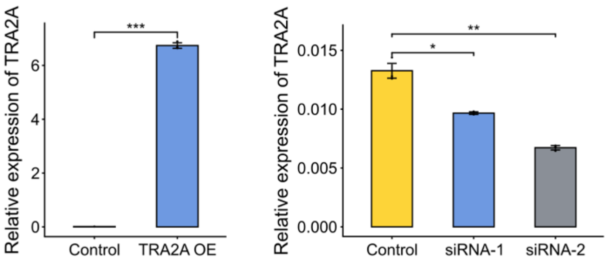

Figure S10. RT-qPCR validation of TRA2A expression following TRA2A KD and OE. Data are presented as means ± s.e.m. Mann-Whitney U tests were performed for statistical analysis (*P < 0.05, **P < 0.01, ***P < 0.001).

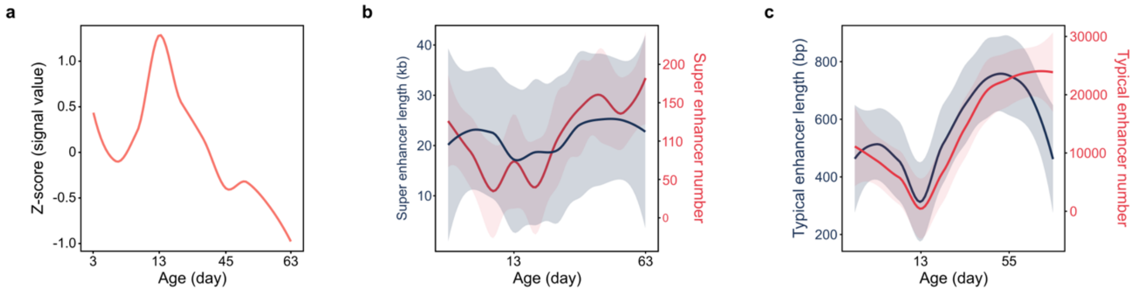

Figure S11. (**a**) Loess regression illustrating age-associated changes in normalized peak signal intensity for H3K27ac across age. (**b**) Age-associated changes in super enhancer length and number across age groups. (**c**) Both typical enhancer length and number show concordant age-associated trends.

Tables

Table S1. Performance metrics of histone modification-based epigenetic clocks across tissues.

Tissue	Histone modification	RMSE	MAE	Pearson's r
Blood	H3K27ac	13.25469	9.95381	0.87956
	H3K27me3	11.74251	9.68004	0.88995
	H3K36me3	14.82734	12.21112	0.83741
	H3K4me1	13.39862	10.56792	0.85497
	H3K4me3	13.32096	10.52806	0.83338
	H3K9me3	17.52021	13.51302	0.78909
Intestine	H3K27ac	4.30575	2.92530	0.93563
	H3K27me3	3.54514	2.71276	0.95631
	H3K36me3	2.02113	1.62990	0.98130
	H3K4me1	3.11860	2.27925	0.97190
	H3K4me3	2.29862	1.31265	0.95212
	H3K9me3	3.48629	2.92209	0.96231
Spleen	H3K27ac	5.12518	4.03650	0.93755
	H3K27me3	6.65258	5.69256	0.82636
	H3K36me3	4.18325	3.74215	0.93267
	H3K4me1	3.57724	2.84529	0.97610
	H3K4me3	4.10956	3.13589	0.92120
	H3K9me3	4.98839	3.88205	0.89155
Heart	H3K27ac	4.94269	2.54003	0.89446
	H3K27me3	6.41097	3.94008	0.87927
	H3K36me3	6.01234	2.72193	0.90110
	H3K4me1	2.82405	2.35321	0.96859
	H3K4me3	3.05505	1.90364	0.95668
	H3K9me3	7.07091	3.29998	0.75879
Liver	H3K27ac	1.53249	1.36004	0.99631
	H3K27me3	6.41827	5.96383	0.95385
	H3K36me3	10.73603	9.12614	0.89244
	H3K4me1	6.66446	6.17267	0.95108
	H3K4me3	2.47663	2.05131	0.99323
	H3K9me3	10.18389	9.28360	0.90499
Stomach	H3K27ac	4.36049	3.07166	0.98766
	H3K27me3	0.57623	0.50094	0.99807
	H3K36me3	2.69371	2.12292	0.95345
	H3K4me1	2.66423	2.47631	0.94309
	H3K4me3	3.75594	3.31927	0.88307
	H3K9me3	4.09453	3.52459	0.91694

Table S2. Filtering metrics for high-quality ChIP-seq datasets.

Metrics[1]	Index	Good	Acceptable
Number of reads (narrow)	Reads	20,000,000	10,000,000
Number of reads (broad)	Reads	45,000,000	45,000,000>Reads>20,000,000
Reads in peaks	RiP%		>0.5%
Non-redundant fraction	NRF	>0.9	0.9>NRF>0.5
PCR-bottlenecking coefficient 1	PBC1	>0.9	0.9>PBC1>0.5
PCR-bottlenecking coefficient 2	PBC2	>10	10>PBC2>1
Normalized strand cross-correlation coefficient	NSC	>1.1	>1.05

[1] QC metrics and thresholds were defined according to ENCODE ChIP-seq guidelines (19).

Table S3. Antibodies used in this study

Name	Manufacturer	Cat #
Monoclonal Anti-Flag M2 antibody produced in mouse	Sigma-Aldrich	#F3165
β-Actin (D6A8) Rabbit mAb	CST	#8457
Phospho-Histone H2A.X (Ser139) Rabbit mAb	CST	#9718
p21 Waf1/Cip1 (12D1) Rabbit mAb	CST	#2947
TNF-α (D5G9) Rabbit mAb	CST	#6945
MMP-3 (D7F5B) Rabbit mAb	CST	#14351
IL-6 (D3K2N) Rabbit mAb	CST	#12153
Histone H3 Antibody	CST	#9715
Goad anti-Rabbit IgG (H+L) Cross-Adsorbed Secondary Antibody, DyLight 594	Invitrogen	#35561
Anti-Histone H3 (acetyl K27) antibody - ChIP Grade	Abcam	#ab4729
Anti-mouse IgG, HRP-linked Antibody	CST	#7076
Anti-rabbit IgG, HRP-linked Antibody	CST	#7074

Table S4. Primers used in this study.

Primer	Sequence (5' - 3')	Comment
TRA2A-pro-F	GATGGCTCCAGAGGTTG	Primers to amplify TRA2A core promoter and TRA2A enhancer
TRA2A-pro-R	CCTAGATAGCGCCACTGC	
TRA2A-enh-F	AGCTTGAATCAGAGGCAAC	
TRA2A-enh-R	TAGCTCCCAATTACTGAGGTC	
FLuc_F	AACACCCCAACATCTTCGAC	Primers to validate the expression of firefly and Renilla luciferase
FLuc_R	TCGCGGTTGTTACTTGACTG	
RLuc_F	TGGCTTCCAAGGTGTACGAC	
RLuc_R	GTTCTCCGCATGTTTCTCGC	
TRA2A-CDS-F	ATGAGTGATGTGGAGGAAAACA	Primers to amplify the complete CDS of TRA2A for overexpression
TRA2A-CDS-R	TCAATAGCGTCTTGGGCTGTA	
Anchor	CGTAGTACATGCACAGATGAGG	Primers for 3C
3C-region1	TCCCGTAGGAGCAATTTGAC	
3C-region2	ATTACAGTGAAGCACAGAGAGG	
3C-region3	ACAGAGCGAGACTCCATCTCC	
3C-region4	TCATCTGCTTCACCTCAGTAAC	
3C-region5	GGCATGTGGTGACCTCGTTT	
3C-region6	CTTCCTTCCTTCCTGGCTAATC	
3C-region7	GGCTAGTATGTCAACTGTGTTAC	
3C-region8	TTGCAGCCATACTCCGTAAC	
3C-region9	AAGGCTGGTCTTGAACTCCT	
3C-region10	GGGTTGTCCTTCACTGATTAAG	
3C-region11	CCACTCCATGAGATCAACAG	
3C-region12	CTGTGATTTAGCTGACAAGCC	
3C-region13	GCCTGGTTGAAACCTAGACTG	
3C-region14	CAGCTATTCAGGAGGCTAAGG	
3C-region15	CCCTCCTCCCATGTTTCATTC	
3C-region16	TACTGGTACTCCCTTCAGTGAC	

3C-region17	GCTGGTCTCAAACTCCTGAC	
3C-region18	GGGAGAGGAGAACAACTGAAAG	
3C-region19	AGGCTAGTCTCGAACTCCTAAC	
3C-region20	GTACACTGCTCGGGTATTGG	
4C-reading primer	TTCACCAGGAAGGGTCCC	Primers for 4C-seq
4C-inverse primer	GAAGGCTGCGTTGGGGTC	
IGF2BP3-qPCR-F	TCGTGACCAGACACCTGATGAG	Primers to assess mRNA expression of IGF2BP3 and TRA2A
IGF2BP3-qPCR-R	GGTGCTGCTTTACCTGAGTCAG	
TRA2A-qPCR-F	AATCGGAGAGCAGGTCAGGATC	
TRA2A-qPCR-R	GAGAATGTCTCCTTGACCTCGAC	
telo-F	CGGTTTGTTTGGGTTTGGGTTTGGGTTTGGGTTTGGGTT	Primers to evaluate relative telomere length
telo-R	GGCTTGCCTTACCCTTACCCTTACCCTTACCCTTACCCT	
36B4-F	CAGCAAGTGGGAAGGTGTAATCC	
36B4-R	CCCATTCTATCATCAACGGGTACAA	
H3K27ac-ChIP-F	TCGCCCTCACCCAGCAAC	Primers to validate H3K27ac enrichment at IGF2BP3 promoter region
H3K27ac-ChIP-R	AAGACTGGCTACGCGTTCGT	

Table S5. Other sequences used in this study.

Name	Sequence (5'-3')	Comment	Manufacturer
sgRNA-1	AGCGCCGTAAATAACGACCG	H3K27ac knockdown target region	Integrated DNA Technologies
sgRNA-2	CCCACCTGAAAGCGCCTCGA		
Stealth siRNA of TRA2A	NA	TRA2A knockdown	Invitrogen (#HSS121028)
MISSION esiRNA of TRA2A	NA		Sigma-Aldrich (EHU137681)

Dataset S1 (separate file). Dataset accessions used to establish histone-based epigenetic clocks.

Dataset S2 (separate file). Contingency tables for Fisher's exact test.

Dataset S3 (separate file). GO biological process enrichment analysis of age-associated DEGs.

Dataset S4 (separate file). GO biological process enrichment analysis of age-associated peaks in heart, intestine, liver, spleen, and stomach.

SI References

1. H. Yin, S. Sweeney, D. Raha, M. Snyder, H. Lin, A high-resolution whole-genome map of key chromatin modifications in the adult Drosophila melanogaster. *PLoS Genet* **7**, e1002380 (2011).
2. H. Li *et al.*, Fly Cell Atlas: A single-nucleus transcriptomic atlas of the adult fruit fly. *Science* **375**, eabk2432 (2022).
3. D. Kim, J. M. Paggi, C. Park, C. Bennett, S. L. Salzberg, Graph-based genome alignment and genotyping with HISAT2 and HISAT-genotype. *Nature Biotechnology* **37**, 907-915 (2019).
4. M. Pertea *et al.*, StringTie enables improved reconstruction of a transcriptome from RNA-seq reads. *Nature Biotechnology* **33**, 290-295 (2015).
5. M. I. Love, W. Huber, S. Anders, Moderated estimation of fold change and dispersion for RNA-seq data with DESeq2. *Genome Biology* **15**, 550 (2014).
6. G. Yu, L. G. Wang, Y. Han, Q. Y. He, clusterProfiler: an R package for comparing biological themes among gene clusters. *Omics* **16**, 284-287 (2012).
7. C. A. Schneider, W. S. Rasband, K. W. Eliceiri, NIH Image to ImageJ: 25 years of image analysis. *Nature Methods* **9**, 671-675 (2012).
8. R. M. Cawthon, Telomere length measurement by a novel monochrome multiplex quantitative PCR method. *Nucleic Acids Res* **37**, e21 (2009).
9. K. Ly *et al.*, Telomere length in early childhood is associated with sex and ethnicity. *Scientific Reports* **9**, 10359 (2019).
10. X. Wang, runHiC: A user-friendly Hi-C data processing software based on hiclib. *Zenodo.* 10.5281/zenodo.55324 (2016).
11. J. Wolff *et al.*, Galaxy HiCExplorer 3: a web server for reproducible Hi-C, capture Hi-C and single-cell Hi-C data analysis, quality control and visualization. *Nucleic Acids Research* **48**, W177-W184 (2020).
12. T. Gao *et al.*, EnhancerAtlas: a resource for enhancer annotation and analysis in 105 human cell/tissue types. *Bioinformatics* **32**, 3543-3551 (2016).
13. L. Lopez-Delisle *et al.*, pyGenomeTracks: reproducible plots for multivariate genomic datasets *Bioinformatics* **37**, 422-423 (2020).
14. P. H. L. Krijger, G. Geeven, V. Bianchi, C. R. E. Hilvering, W. de Laat, 4C-seq from beginning to end: A detailed protocol for sample preparation and data analysis. *Methods* **170**, 17-32 (2020).
15. Z. Huang, C. Wang, E. Treuter, R. Fan, An optimized 4C-seq protocol based on cistrome and epigenome data in the mouse RAW264.7 macrophage cell line. *STAR Protoc* **3**, 101338 (2022).
16. S. Thongjuea, R. Stadhouders, F. G. Grosveld, E. Soler, B. Lenhard, r3Cseq: an R/Bioconductor package for the discovery of long-range genomic interactions from chromosome conformation capture and next-generation sequencing data. *Nucleic Acids Res* **41**, e132 (2013).
17. N. Naumova, E. M. Smith, Y. Zhan, J. Dekker, Analysis of long-range chromatin interactions using Chromosome Conformation Capture. *Methods* **58**, 192-203 (2012).

18. H. Hagège *et al.*, Quantitative analysis of chromosome conformation capture assays (3C-qPCR). *Nat Protoc* **2**, 1722-1733 (2007).
19. S. G. Landt *et al.*, ChIP-seq guidelines and practices of the ENCODE and modENCODE consortia. *Genome Res* **22**, 1813-1831 (2012).
