# Supplementary material for: Histone modification clocks for robust cross-species biological age prediction and elucidating senescence regulation
Source: Proc Natl Acad Sci U S A. 2026 Mar 10;123(11):e2533687123. doi: 10.1073/pnas.2533687123 (PMC12993953; doi:10.1073/pnas.2533687123)

GO biological process enrichment of age-associated peaks in the heart

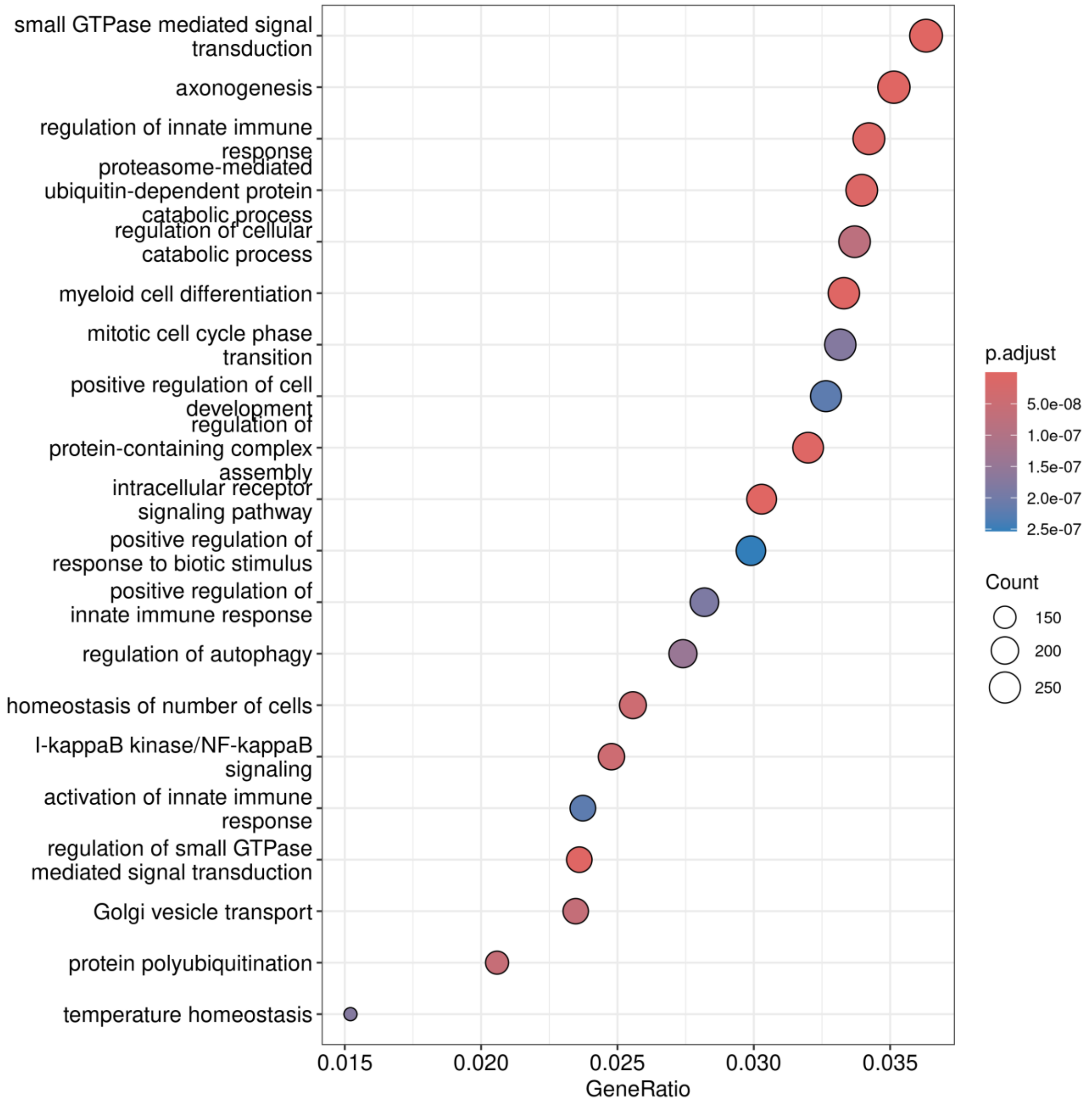

GO biological process enrichment of age-associated peaks in the intestine

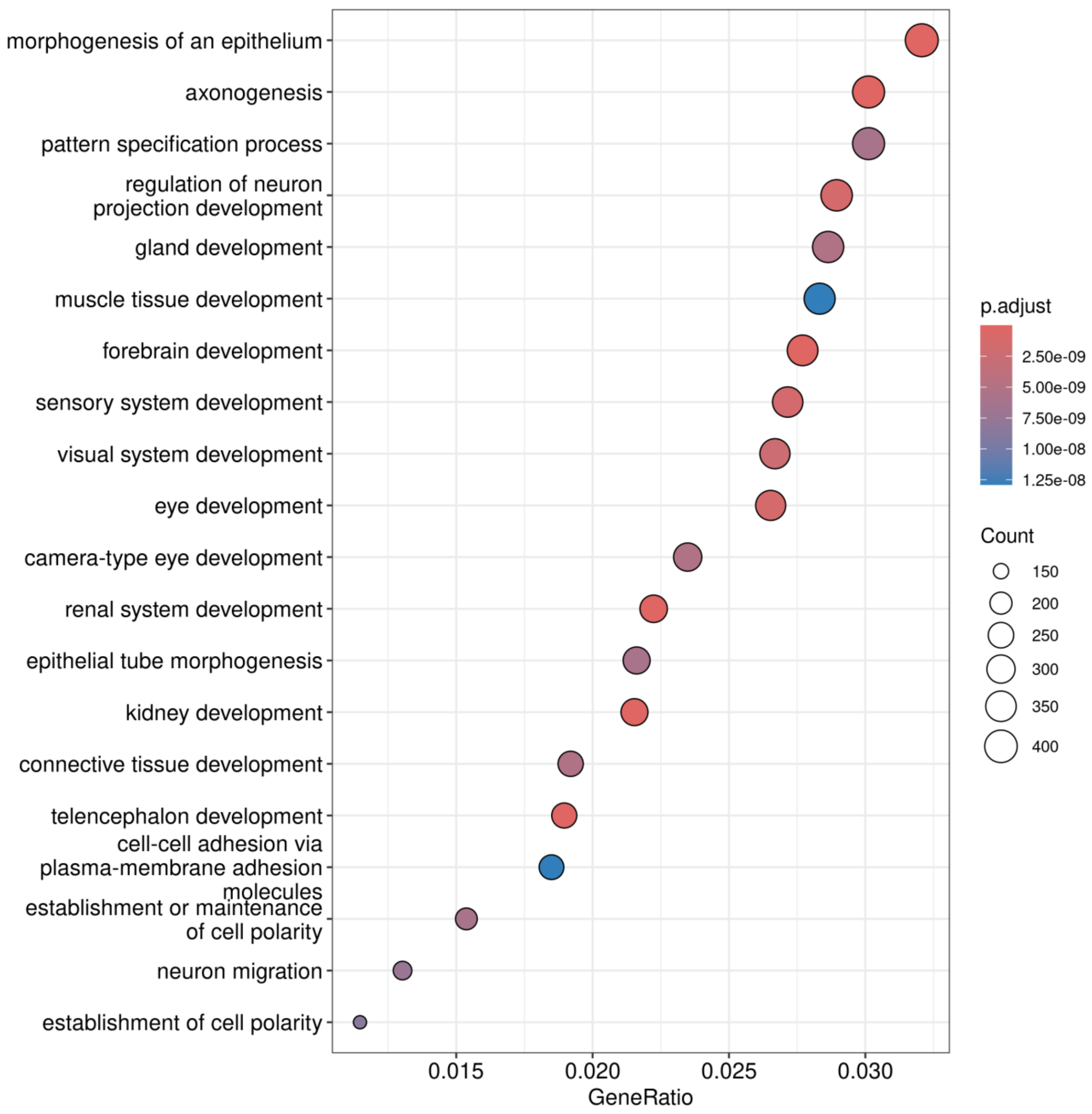

GO biological process enrichment of age-associated peaks in the liver

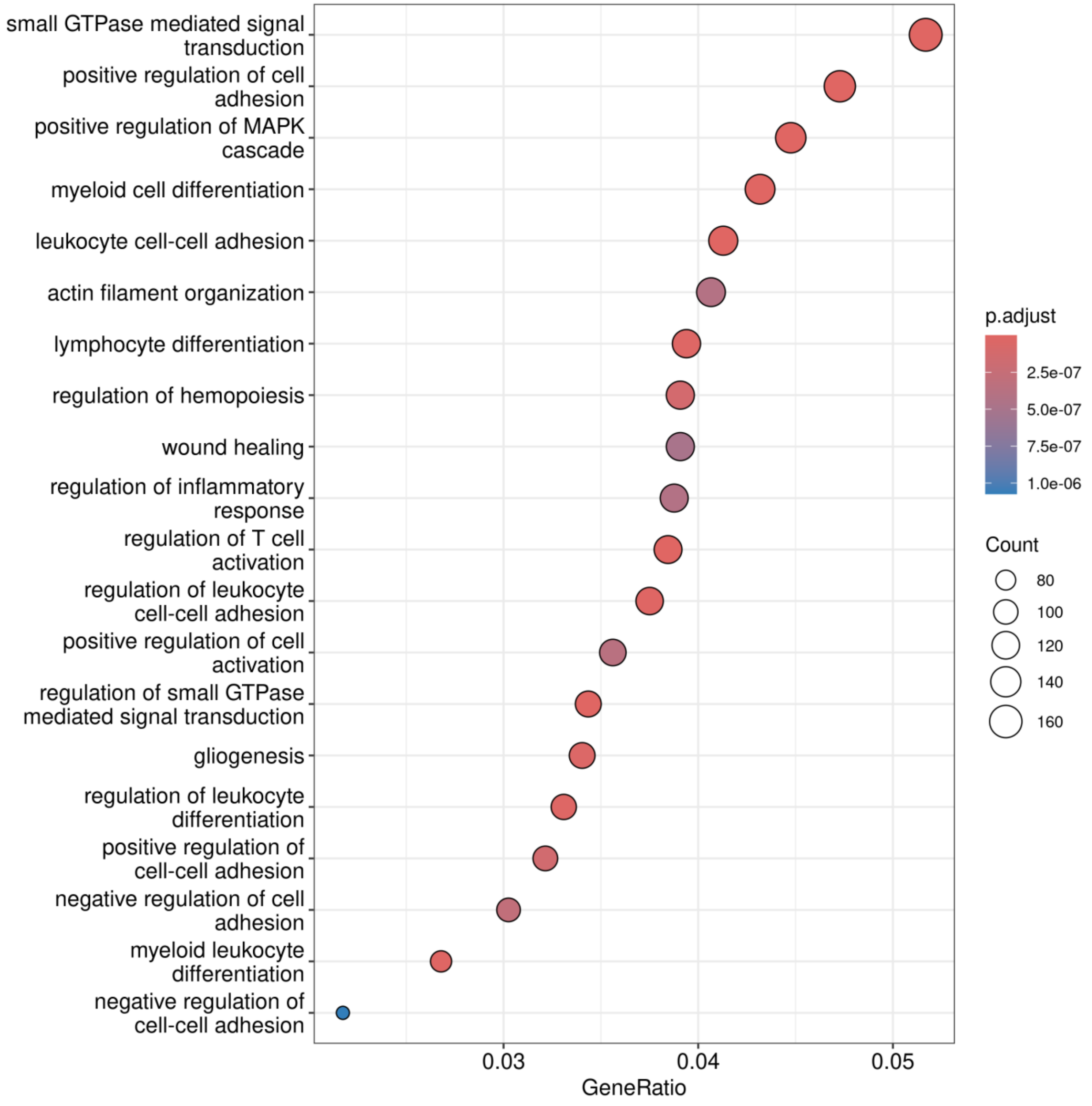

GO biological process enrichment of age-associated peaks in the spleen

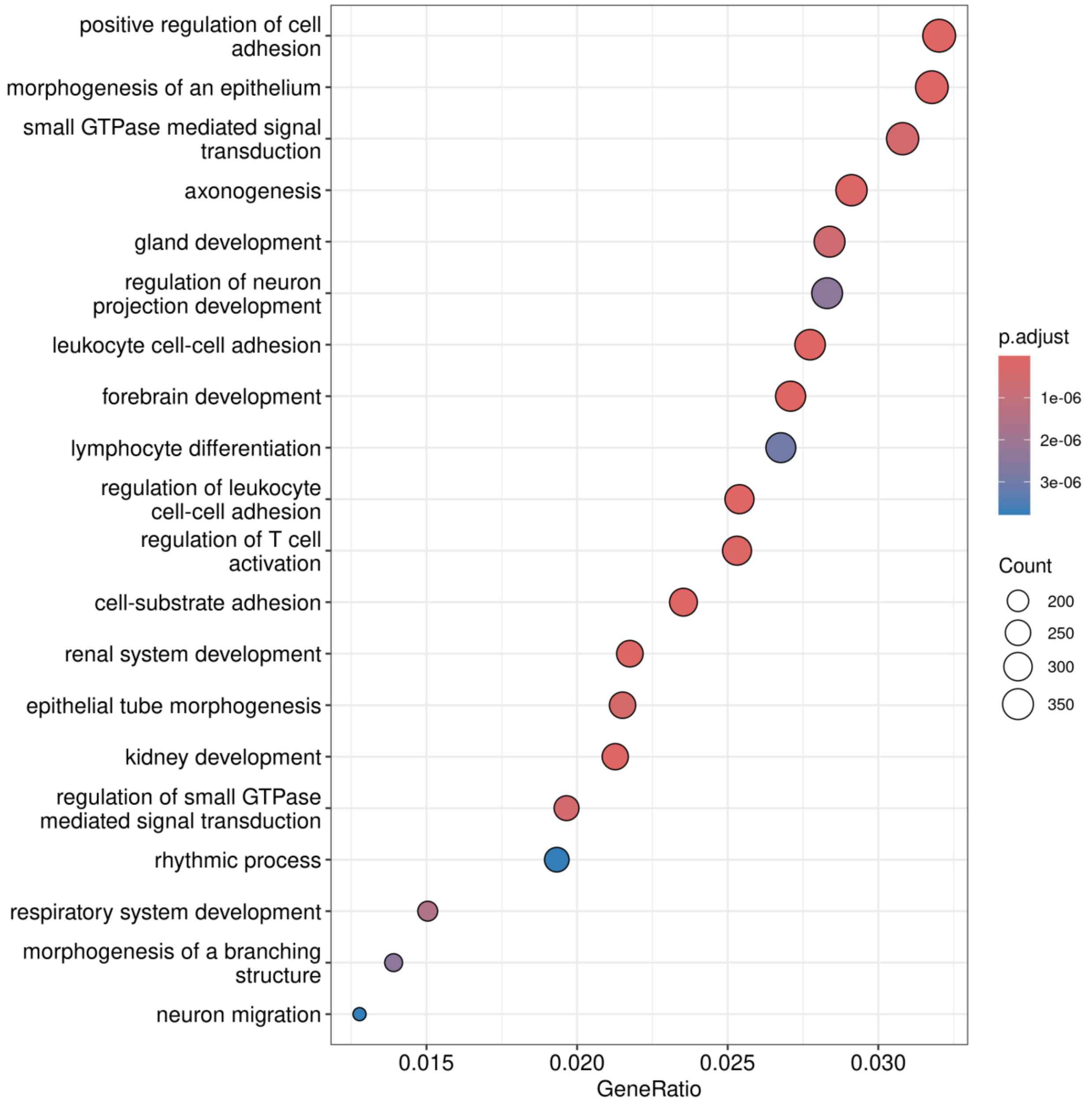

GO biological process enrichment of age-associated peaks in the stomach

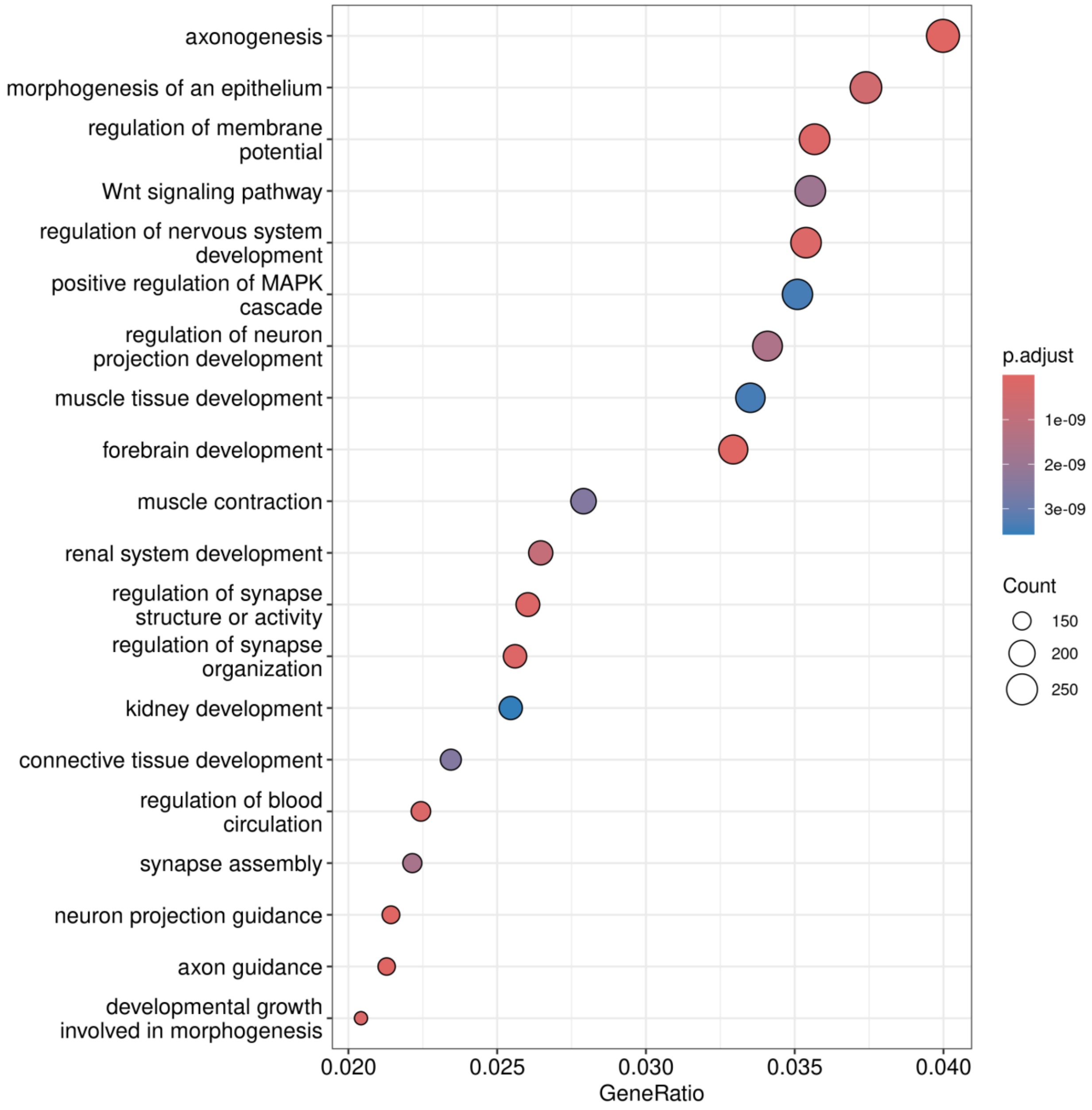

Supplement: Supplementary file 5 — Dataset S04 (PDF) [file pnas.2533687123.sd04.pdf]
